# Ferroptosis Patterns and Tumor Microenvironment Infiltration Characterization in Bladder Cancer

**DOI:** 10.3389/fcell.2022.832892

**Published:** 2022-03-21

**Authors:** Qi-Dong Xia, Jian-Xuan Sun, Chen-Qian Liu, Jin-Zhou Xu, Ye An, Meng-Yao Xu, Zheng Liu, Jia Hu, Shao-Gang Wang

**Affiliations:** Department and Institute of Urology, Tongji Hospital, Tongji Medical College, Huazhong University of Science and Technology, Wuhan, China

**Keywords:** ferroptosis, tumor microenvironment, tumor mutation burden, bladder cancer, immunotherapy

## Abstract

**Background:** Ferroptosis is a unique iron-dependent form of cell death and bladder cancer (BCa) is one of the top ten most common cancer types in the world. However, the role of ferroptosis in shaping the tumor microenvironment and influencing tumor clinicopathological features remains unknown.

**Methods:** Using the data downloaded from The Cancer Genome Atlas (TCGA) and Gene Expression Omnibus (GEO), we comprehensively evaluated the ferroptosis patterns of 570 BCa samples based on 234 validated ferroptosis genes reported in the FerrDb database and systematically correlated these ferroptosis patterns with tumor microenvironment (TME) cell-infiltrating characteristics. The ferroptosis score was constructed to quantify ferroptosis patterns of individuals using principal component analysis (PCA) algorithms.

**Results:** Four distinct ferroptosis patterns and two gene clusters were finally determined. Significant differences in clinical characteristics and the prognosis of patients were found among different ferroptosis patterns and gene clusters, so were in the mRNA transcriptome and the landscape of TME immune cell infiltration. We also established a set of scoring system to quantify the ferroptosis pattern of individual patients with BCa named the ferroptosis score, which was discovered to tightly interact with clinical signatures such as the TNM category and tumor grade and could predict the prognosis of patients with BCa. Moreover, tumor mutation burden (TMB) was positively correlated to the ferroptosis score, and the low ferroptosis score was related to a better response to immunotherapy using PD-1 blockade. Finally, we also found there existed a positive correlation between the sensitivity to cisplatin chemotherapy and ferroptosis score.

**Conclusions:** Our work demonstrated and interpreted the complicated regulation mechanisms of ferroptosis on the tumor microenvironment and that better understanding and evaluating ferroptosis patterns could be helpful in guiding the clinical therapeutic strategy and improving the prognosis of patients with BCa.

## Introduction

Ferroptosis is a unique iron-dependent form of cell death and is morphologically, biochemically, and genetically distinct from apoptosis, necrosis, pyroptosis, and autophagy (Dixon et al., 2012). Abundant and accessible cellular iron is necessary to induce ferroptosis (Dixon et al., 2012). The first discovered ferroptosis inducers are erastin (Dolma et al., 2003) and RSL3 (Yang and Stockwell, 2008), and then, a variety of ferroptosis inducers have been found in succession such as sorafenib, sulfasalazine, FIN56, and so on (Liang et al., 2019). System Xc^−^ inhibition and glutathione (GSH) peroxidase 4 (GPX4) inhibition are the two main mechanisms that induce ferroptosis (Liang et al., 2019). System Xc^−^ is the glutamate/cystine antiporter which can facilitate the exchange of cystine and glutamate across the plasma membrane (Bridges et al., 2012). The inhibition of system Xc^−^ can decrease the intracellular cysteine level, which is the precursor for glutathione synthesis (Yang et al., 2014). GPX4 is an indispensable enzyme which catalyzes the reduction of lipid hydroperoxide within a complex cellular membrane environment, which utilizes glutathione as an essential cofactor for its enzymatic activity (Brigelius-Flohé and Maiorino, 2013). Therefore, the inhibition of both system Xc^−^ and GPX4 will result in the accumulation of iron-dependent lipid hydroperoxides and increased level of reactive oxygen species (ROS), and finally, lead to cell death (Chan et al., 2019). Ferroptosis is regulated by several molecular pathways such as the transsulfuration pathway and mevalonate pathway (Yang and Stockwell, 2016), and a variety of ferroptosis regulators participate in these pathways (Liang et al., 2019). In recent years, ferroptosis has been discovered to be related to many human diseases such as acute kidney injury, Huntington disease, periventricular leukomalacia, and so on, among which cancer was the one that researchers paid most attention to (Liang et al., 2019). Different cancers seem to exhibit significantly different susceptibility to ferroptosis (Mou et al., 2019); therefore, increasing the sensitivity to ferroptosis and developing new therapies targeted at ferroptosis could be an intriguing and challenging research field in the future.

Bladder cancer (BCa) is one of the top ten most common cancer types in the world and accounted for approximately 550,000 new cases and 200,000 deaths in 2018 (Richters et al., 2020). In the United States alone, in 2019, the number of new cases and deaths were 80,470 and 17,670, respectively (Siegel et al., 2019). Advanced age, male sex, tobacco smoking, and occupational exposure to some chemical agents are the main risk factors for the incidence of BCa (Sylvester et al., 2021). According to the depth of tumor invasion and infiltration, BCa can be divided into non-muscle invasive bladder cancer (NMIBC) and muscle invasive bladder cancer (MIBC). Patients with NMIBC are treated with endoscopic resection and adjuvant intravesical therapy, including intravesical chemotherapy and intravesical bacillus Calmette–Guérin (BCG) immunotherapy (Sylvester et al., 2021). Patients with MIBC can choose radical cystectomy (RC) and lymphadenectomy, pre- and post-operative radiotherapy, neoadjuvant immunotherapy, as well as chemotherapy depending on the risk classification (Witjes et al., 2021). However, the efficacy of various treatments for BCa remains unideal and new therapeutic strategies need to be developed.

Recently, several studies have focused on the interaction between ferroptosis and BCa. A variety of ferroptosis-related signatures were established based on ferroptosis regulator genes (FRGs) to predict the landscape of the epithelial-mesenchymal transition (EMT) status, the tumor microenvironment (TME), and the prognosis of patients with BCa, and it seems that these signatures had their unique roles in evaluating their response to chemotherapy and immunotherapy (Cui et al., 2021; Sun et al., 2021; Yan et al., 2021). However, the establishment of these signatures was confined to limited ferroptosis regulator genes and many other novel validated ferroptosis-related genes were ignored. So, in this article, we evaluated the genetic variation of 23 ferroptosis regulators in BCa among a total of 412 samples from the TCGA-BLCA cohort, explored the FerrDb database to find validated ferroptosis genes (VFGs), divided the patients with BCa into four ferroptosis patterns according to the expression levels of VFGs and performed survival analysis, and then we further explored the TME cell infiltration characteristics in distinct VFG patterns and surprisingly found VFG patterns were tightly connected with TME. Next, we discovered 367 VFG cluster-related differentially expressed genes (DEGs), classified the patients into two distinct genomic subgroups, and explored the interaction between VFG patterns and gene patterns. Finally, we established a set of scoring system termed the ferroptosis score to quantify the ferroptosis pattern in individual patients and explored the characteristics of ferroptosis in tumor somatic mutation, immunotherapy, and chemotherapy.

## Materials and Methods

### Data Retrieval and Processing

Ferroptosis regulator genes (FRGs) were obtained from Liu et al. (2020). Ferroptosis-related genes with validated evidences were obtained from FerrDb (http://www.zhounan.org/ferrdb/). RNA-sequencing data of BCa patients were searched from The Cancer Genome Atlas (TCGA database, https://portal.gdc.cancer.gov/) and the Gene Expression Omnibus (GEO database, https://www.ncbi.nlm.nih.gov/gds/?term=). Notably, datasets without detailed corresponding survival information or with a small sample size were excluded. Finally, two eligible high-quality bulk-seq cohorts, TCGA_BLCA and GSE13507, were enrolled in this study. Among them, transcriptome profiles in TCGA_BLCA datasets were downloaded in the fragments per kilobase of transcript per million mapped reads (FPKM) format. Then, we transformed the FPKM values of each sample into transcripts per kilobase million (TPM) values. Normalized matrix files with corresponding clinical information of GSE13507 were downloaded. We merged these two datasets and used the combat algorithm to eliminate the batch effects by R package “sva”. The mutation atlas was also downloaded from the TCGA database. The copy number variation matrix was obtained from UCSC-Xena (http://xena.ucsc.edu/). All the original data were processed and analyzed by the R program version 4.1.1.

### Differential Expression Status, Mutation Atlas, and Copy Number Variation of FRGs

We first systematically investigated the expression, mutant, copy number variant status of the FRGs. Differentially expressed analysis was performed between normal tissue and tumor tissue in both TCGA_BLCA and GSE13507. The mutation atlas of these FRGs was extracted from the original matrix and visualized. The copy number variation atlas was annotated and visualized in the genome cycle plot.

### Role of FRGs in Bladder Cancer and the Unsupervised Cluster of VFGs

Having merged the gene matrix and eliminating the batch effects, we performed both univariate cox regression and log-rank test to check the prognostic value of the FRGs. Then, we performed the Spearman correlation test between every two FRGs to investigate the co-expression status between FRGs. Subsequently, we conducted an unsupervised cluster of all the VFGs in FerrDb, and the number of clusters was determined according to the algorithm of consensus clustering. Notably, this unsupervised clustering was conducted by R package “ConsensusClusterPlus”, and all the procedures were repeated 1,000 times to ensure and verify the stability of the unsupervised cluster.

### Survival Differences, Potential Functions, and Immune Infiltrations of the VFG Clusters

Having obtained the classification clustered by the VFG, we performed survival analysis to investigate the survival differences between VFG clusters. Kaplan–Meier survival curves were plotted, and the log-rank test was conducted. Principle components analysis (PCA) was applied to check the discrimination between different VFG clusters. In addition, gene set variation analysis (GSVA) was used to compare the differential enhanced functions or pathways between different VFG clusters. Finally, single sample gene set enrichment analysis (ssGSEA) was used to estimate the immune infiltration and immune-related functions of each sample. The Wilcoxon test was applied to compare the differential immune infiltration and immune-related functions between VFG clusters.

### Differentially Expressed Genes Between VFG Clusters and the Establishment of the Ferroptosis Score

Differentially expressed analysis was performed between every two VFG clusters to seek the DEGs. Subsequently, we took an intersect of these DEGs in different comparisons and obtained the final DEGs. GO enrichment analysis and KEGG enrichment analysis were conducted to further investigate the potential functions and mechanisms of these DEGs. Subsequently, univariate cox regression was carried out to seek those DEGs with prognostic value. Following this, an unsupervised cluster was performed again based on the left DEGs to quantify the detailed ferroptosis patterns in BCa patients. Then, we performed principal component analysis (PCA) to distinguish the molecular characteristics of these DEGs with prognostic value and obtained a ferroptosis score formula according to the PCA:

Ferroptosis score=∑(PC1+PC2).

Among the formula, PC1 and PC2 separately mean the expression score in two dimensions of the DEGs. Thus, the sum of these two scores was named the ferroptosis score, which can represent the ferroptosis patterns to some extent.

### Further Verification and Functional Investigation of the Ferroptosis Score

In all included samples with detailed survival information, we set the threshold according to the best cut-off value in the TCGA_ BLCA cohort calculated by the “surv_cutpoint” function in R package “survminer”. Here, the best cut-off is -0.0410544, and then all patients including TCGA_ BLCA and GSE13507 cohorts were divided into high or low ferroptosis score groups. Cohorts that are higher than -0.0410544 are defined as the high ferroptosis score group and those that are lower are the low ferroptosis score group. Survival analysis in all patients, TCGA_BLCA cohort, and GSE13507 cohort, was separately conducted to check whether this ferroptosis score was associated with survival. Then we divided all patients into several sub-groups according to their clinicopathological characteristics, including age, gender, grade, T stage, N stage, and M stage. Then survival analysis was applied in each sub-group to investigate the universality of this ferroptosis score. In addition, in the TCGA_BLCA cohort, we calculated the tumor mutation burden (TMB) of each patient according to its somatic mutation profiles. Then we further investigated the correlation between TMB and the ferroptosis score, and combined these two factors to predict the survival of patients with BCa. Following this, we separately summarized the mutation atlas of the patients with low-/high ferroptosis scores and compared the mutant frequencies of each gene between the high ferroptosis score group and low ferroptosis score group by the χ^2^ test.

More importantly, as the biological process of ferroptosis is associated with both chemotherapy and immunotherapy, we further explored the association between the drug sensitivity to chemotherapy (cisplatin, doxorubicin, methotrexate, vinblastine) which was predicted by the R package “pRRophetic” (Geeleher et al., 2014). Notably, here we performed three methods to predict the response to immunotherapy: TCIA (Charoentong et al., 2017), TIDE (Jiang et al., 2018), and submap (Hoshida et al., 2007). The drug response to chemotherapy and immunotherapy was compared between the high- or low-ferroptosis score groups by the Wilcoxon test or χ^2^ test. In addition, we calculated the ferroptosis score of each patient in IMvigor-210 cohort to externally validate the predicted response to immunotherapy. Response to anti-PD-L1 immunotherapy in IMvigor-210 cohort was also compared between the high- or low-ferroptosis score groups.

### Statistical Analysis

All the data processing and statistical analysis were conducted by R software version 4.1.1. All the *p*-values were on two sides, and *p*-value< 0.05 was considered with statistical significance.

## Results

### Landscape of Genetic Variation of Ferroptosis Regulators in Bladder Cancer

In this study, 23 genes were identified to play critical roles in regulating ferroptosis and were defined as ferroptosis regulator genes (FRGs), including cyclin-dependent kinase inhibitor 1 (CDKN1A), nuclear factor, erythroid 2 like 2 (NFE2L2), Fanconi anemia complementation group D2 (FANCD2), transferrin receptor (TFRC), dipeptidyl-dippeptidase-4 (DPP4), heat shock protein family A member 5 (HSPA5), lysophosphatidylcholine acyltransferase 3 (LPCAT3), cysteinyl tRNA synthetase (CARS), nuclear receptor coactivator 4 (NCOA4), citrate synthase (CS), arachidonate 15-lipoxygenase (ALOX15), ribosomal protein L8 (RPL8), glutaminase 2 (GLS2), solute carrier family 7 member 11 (SLC7A11), heat shock protein beta 1 (HSPB1), solute carrier family 1 member 5 (SLC1A5), acyl-CoA synthetase long-chain family member 4 (ACSL4), ER membrane protein complex subunit 2 (TTC35/EMC2), metallothionein-1G (MT1G), glutathione peroxidase 4 (GPX4), CDGSH iron sulfur domain 1 (CISD1), spermidine/spermine N1-acetyl-transferase 1 (SAT1), and ATP synthase membrane subunit C locus 3 (ATP5MC3/ATP5G3) (Stockwell et al., 2017). Among the 23 FRGs, 10 are negative regulators, including CDKN1A, HSPA5, EMC2, SLC7A11, NFE2L2, MT1G, HSPB1, GPX4, FANCD2, and CISD1, and 13 are positive regulators, including SLC1A5, SAT1, TFRC, RPL8, NCOA4, LPCAT3, GLS2, DPP4, CS, CARS, ATP5MC3, ALOX15, and ACSL4.

**FIGURE 1:**
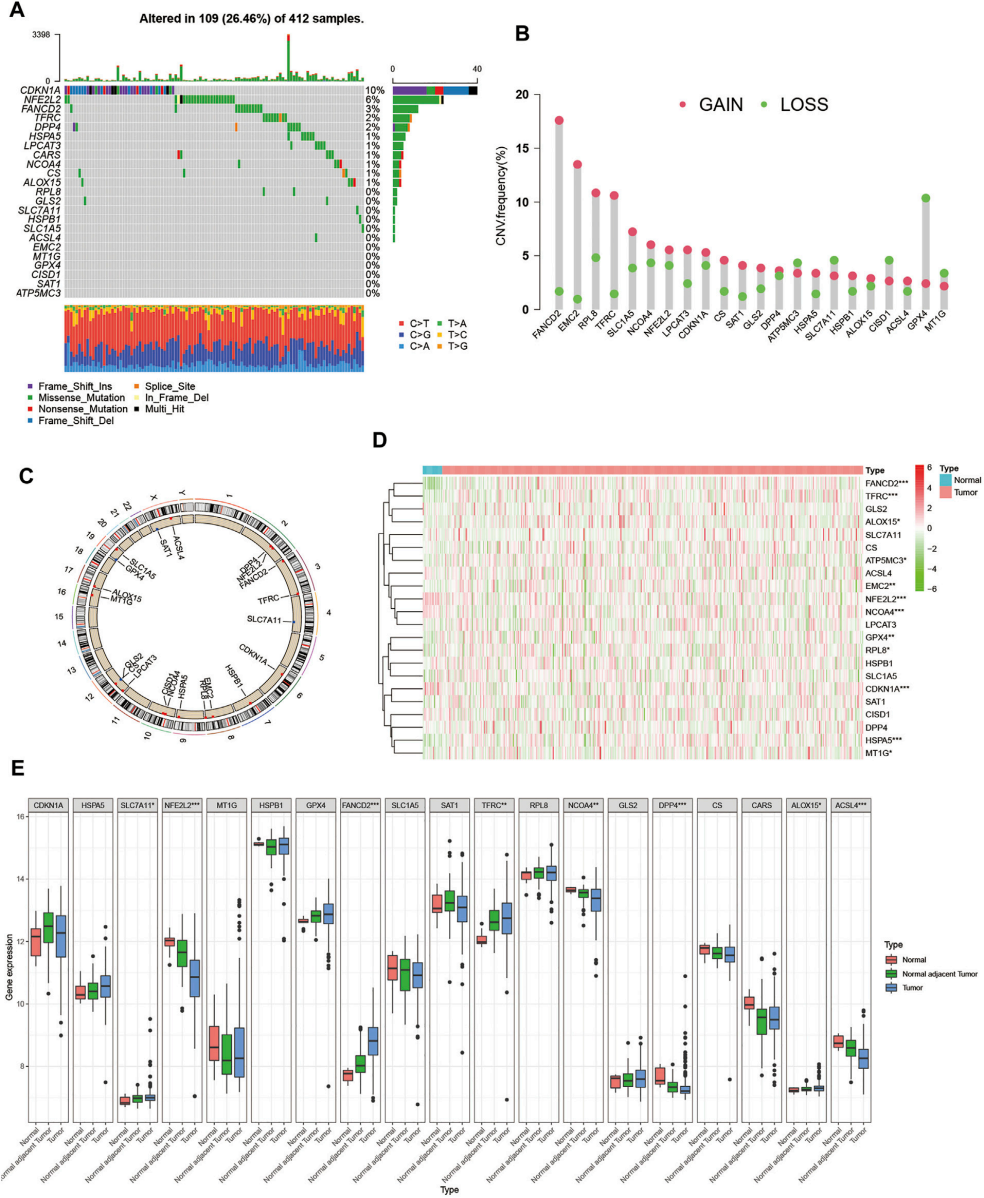
Landscape of genetic and expression variation of ferroptosis regulators in BCa. **(A)** Mutation frequency of 23 ferroptosis regulator genes in 412 patients with BCa from the TCGA-BLCA cohort. Each column represented individual patients. The upper barplot showed TMB. The number on the right indicated the mutation frequency in each regulator gene. The right barplot showed the proportion of each variant type. The stacked barplot below showed a fraction of conversions in each sample. **(B)** CNV variation frequency of ferroptosis regulator genes in GSE13507 cohort. The height of the column represented the alteration frequency. The deletion frequency, green dot; The amplification frequency, red dot. **(C)** Location of CNV alteration of ferroptosis regulator genes on 23 chromosomes using GSE13507 cohort. **(D)** Expression of 22 ferroptosis regulator genes between normal bladder tissues and tumor tissues in the TCGA-BLCA cohort. Tumor, red; Normal, blue. Each column represented individual samples. The upper line represented the type of tissues. The color of each pane represented the expression level. **(E)** Expression of 19 ferroptosis regulator genes between normal bladder tissues, normal adjacent tumor tissues and tumor tissues in GSE13507 cohort. Tumor, blue; Normal adjacent tumor, green; Normal, red. The upper and lower ends of the boxes represented interquartile range of values. The lines in the boxes represented median value, and black dots showed outliers. The asterisks represented the statistical *p* value (*p < 0.05; **p < 0.01; ***p < 0.001).

First, we summarized the incidence of copy number variations (CNV) and somatic mutations of the 23 FRGs in BCa. Mutations of FRGs occurred in 109 samples among a total of 412 samples from the TCGA-BLCA cohort with a frequency of 26.46%. We found that CDKN1A exhibited the highest mutation frequency followed by NFE2L2 and FANCD2, while EMC2, MT1G, GPX4, CISD1, SAT1, and ATP5MC3 showed no mutation in BCa samples (Figure 1A). Since CDKN1A had the highest mutation frequency, we then explored whether mutations in CDKN1A would influence the expression of other FRGs. As shown in Supplementary Figure S1, the expression levels of other FRGs were remarkably different between CDKN1A mutation and wild samples, among which GLS2 and TFRC expression were higher in CDKN1A mutation samples while MT1G and RPL8 were just opposite (Supplementary Figures S1A–D). Then, we investigated the CNV alteration in 23 FRGs and found a prevalent CNV alteration in 22 FRGs, among which most alterations were gained in copy number, while ATP5MC3, SLC7A11, CISD1, GPX4, and MT1G had a greater frequency of CNV loss (Figure 1B). The location of CNV alteration of FRGs on chromosomes was shown in Figure 1C. Next, we searched the GEO and TCGA databases for public gene expression data in tumor, normal adjacent tumor tissue, and normal tissue to find whether the above genetic variations could influence the FRGs’ expression in BCa patients. We discovered that both mutation and CNV alteration contributed to the difference in expression levels of FRGs but CNV alteration might play a more critical role. Most FRGs with gain of CNV exhibited significantly higher expression in BCa tumor tissues compared to normal adjacent tumor tissues or normal bladder tissues, such as FANCD2, EMC2, and TFRC. But when it came to FRGs with loss of CNV, the differences between tumor tissue and normal adjacent tumor tissue or normal bladder tissue were inconsistent with the variation in CNV, which indicated that there existed other ways of regulating the expression of FRGs except for CNV variation (Figures 1D,E). The above analyses showed the high heterogeneity of the genetic and expression alteration landscape of FRGs between normal and BCa samples, indicating that the expression imbalance of FRGs played a crucial role in the occurrence and progression of BCa.

### Validated Ferroptosis Gene Patterns Classified According to the Expression of Validated Ferroptosis Genes

**FIGURE 2:**
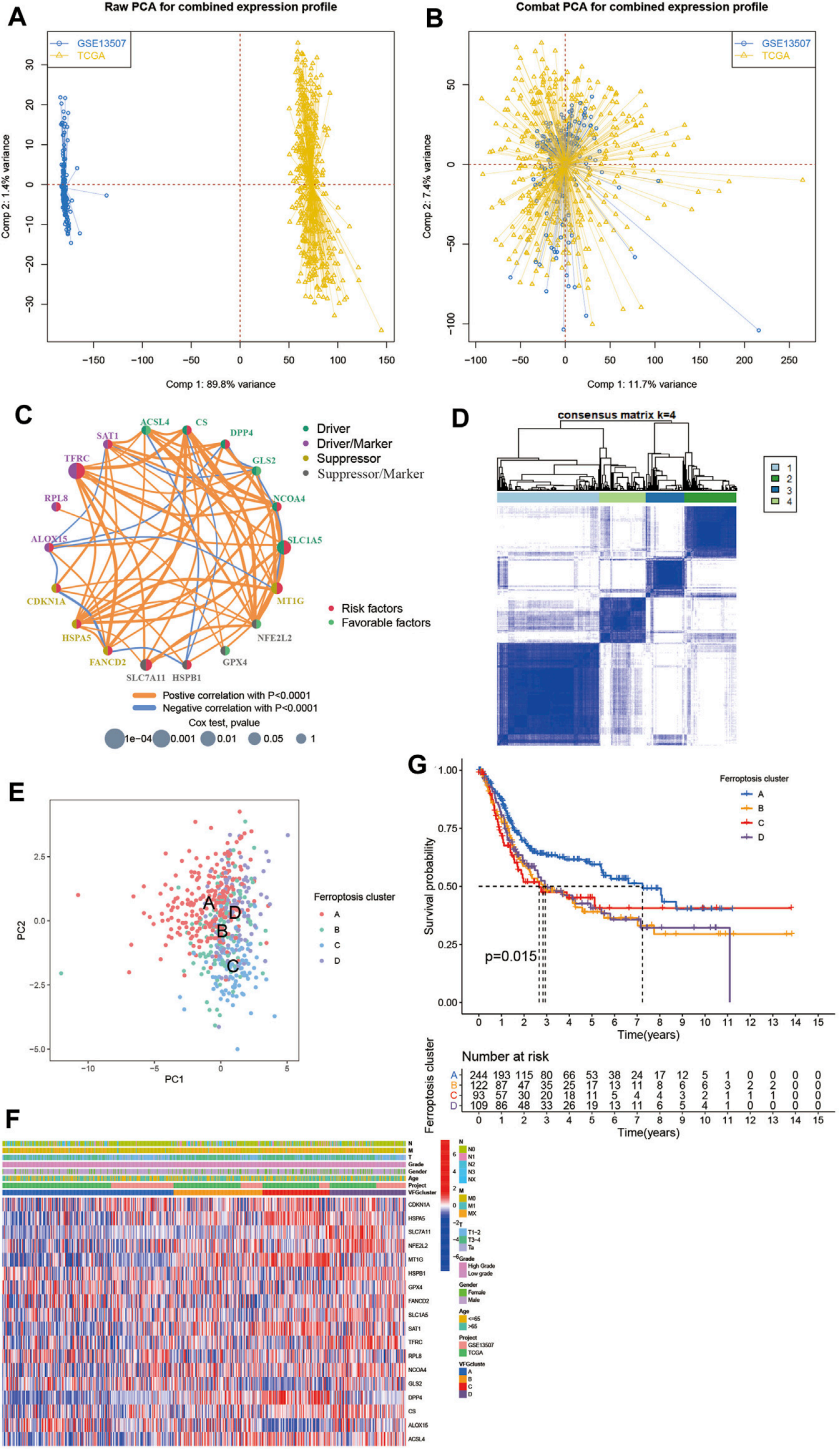
Patterns of ferroptosis and biological characteristics of each pattern. **(A,B)** Principal component analysis for the expression profiles of common genes before and after combination of GSE13507 and the TCGA-BLCA cohort. Before processing, two subgroups without intersection were identified, indicating the GSE13507 and TCGA-BLCA samples were well distinguished based on the expression profiles of their common genes, while the two datasets merged together well after processing. Samples from GSE13507 were marked with blue and samples from TCGA-BLCA marked with yellow. **(C)** Interaction between FRGs in BCa. The circle size represented the effect of each ferroptosis regulator gene on the prognosis, and the range of values calculated by Log-rank test was *p* < 0.0001, *p* < 0.001, *p* < 0.01, *p* < 0.05 and *p* < 0.1, respectively. Red dots in the circle, risk factors of prognosis; Light green dots in the circle, protective factors of prognosis. The lines linking regulators showed their interactions, and their thickness showed the correlation strength between regulator genes. Negative correlation was marked with blue and positive correlation with orange. The driver, driver/marker, suppressor, suppressor/marker were marked with dark green, purple, yellow, and gray, respectively. **(D)** Consensus matrices of the meta-cohort for k = 4. **(E)** Principal component analysis for the transcriptome profiles of four ferroptosis patterns, showing a remarkable difference on transcriptome between different ferroptosis patterns. **(F)** Unsupervised clustering of 234 validated ferroptosis genes in the meta-cohort. The VFGcluster, project, age, gender, grade, and TNM category were used as patient annotations. Red represented high expression of regulators and blue represented low expression. **(G)** Kaplan–Meier curves indicated ferroptosis patterns were markedly related to overall survival of 568 patients in meta-cohort, of which 244 cases were in VFGcluster A, 122 cases in VFGcluster B, 93 cases in VFGcluster C, and 109 cases in VFGcluster D (*p* = 0.015, Log-rank test).

To further investigate the interaction between ferroptosis genes and tumor characteristics, we explored the FerrDb database established by Zhou et al. which collected nearly all the ferroptosis regulators and markers reported in published articles from PubMed up to 20 February 2020 (Zhou and Bao, 2020). In this database, genes were annotated as drivers, suppressors, and markers according to their function reported in the original article. And the confidence level was divided into four categories sorted by experimental reliability and reproducibility: validated, screened, predicted, and deduced (Zhou and Bao, 2020). In Table 1, we summarized the 23 FRGs according to their annotations in the FerrDb database. Next, we used Combat R packages to eliminate the heterogeneity between the GEO dataset GSE13507 and TCGA-BLCA cohort and enrolled them into a new meta-cohort. Before processing, we could easily distinguish the two datasets by principal component analysis (PCA) (Figure 2A), while the two datasets merged well together after processing (Figure 2B). The detailed characteristics of the included patients were shown in Table 2. Then we divided the meta-cohort into two subgroups according to the expression level of every FRG and performed survival analysis (Supplementary Figures S2A–N). As shown in the figures, the survival outcomes were significantly associated with the expression levels of the fourteen FRGs, among which higher expression level of ACSL4 and GPX4 predicted a better prognosis while lower expression level of ALOX15, CDKN1A, DPP4, FANCD2, HSPA5, HSPB1, MT1G, NCOA4, RPL8, SLC1A5, SLC7A11, and TFRC revealed a survival advantage. The comprehensive landscape of FRGs’ interactions, connections, and their prognostic significance for BCa patients was described in the FRG network (Figure 2C). In the network we could find that not only FRGs in the same categories (e.g., drivers and drivers, suppressors and suppressors) exhibited a significant correlation, but also a remarkable correlation was shown between drivers, suppressors, and markers. The results uncovered the latent cross-talk among the FRGs which might play a crucial role in the prognosis of BCa patients and needed to be further studied.

**TABLE 1:** Annotation of 23 FRGs in the FerrDb database.

FRG	Type	Confidence
CDKN1A	Suppressor	Validated
HSPA5	Suppressor	Validated
EMC2	Driver	Validated
SLC7A11	Suppressor/Marker	Validated
NFE2L2	Suppressor/Marker	Validated
MT1G	Suppressor	Validated
HSPB1	Suppressor/Marker	Validated
GPX4	Suppressor/Marker	Validated
FANCD2	Suppressor	Validated
CISD1	Suppressor	Validated
SLC1A5	Driver	Validated
SAT1	Driver/Marker	Validated
TFRC	Driver/Marker	Validated
RPL8	Driver/Marker	Validated
NCOA4	Driver	Validated
LPCAT3	Driver	Validated
GLS2	Driver	Validated
DPP4	Driver	Validated
CS	Driver	Validated
CARS	Driver	Validated
ATP5MC3	Driver/Marker	Validated/Deduced
ALOX15	Driver/Marker	Validated/Deduced
ACSL4	Driver	Validated

**TABLE 2:** Basic characteristics of the included patients.

	Overall	GSE13507	TCGA	*p*
n	568	165	403	
Status = Alive/Dead (%)	323/245 (56.9/43.1)	96/69 (58.2/41.8)	227/176 (56.3/43.7)	0.755
Age (mean (SD))	67.22 (11.08)	65.18 (11.97)	68.06 (10.60)	0.005
Gender = Female/Male (%)	135/433 (23.8/76.2)	30/135 (18.2/81.8)	105/298 (26.1/73.9)	0.058
Grade (%)				<0.001
High Grade	440 (77.5)	60 (36.4)	380 (94.3)	
Low Grade	125 (22.0)	105 (63.6)	20 (5.0)	
Unknown	3 (0.5)	0 (0.0)	3 (0.7)	
T (%)				<0.001
T1	83 (14.6)	80 (48.5)	3 (0.7)	
T2	149 (26.2)	31 (18.8)	118 (29.3)	
T3	210 (37.0)	19 (11.5)	191 (47.4)	
T4	69 (12.1)	11 (6.7)	58 (14.4)	
Ta	24 (4.2)	24 (14.5)	0 (0.0)	
Unknown	33 (5.8)	0 (0.0)	33 (8.2)	
M (%)				<0.001
M0	351 (61.8)	158 (95.8)	193 (47.9)	
M1	18 (3.2)	7 (4.2)	11 (2.7)	
MX	197 (34.7)	0 (0.0)	197 (48.9)	
Unknown	2 (0.4)	0 (0.0)	2 (0.5)	
N (%)				<0.001
N0	385 (67.8)	151 (91.5)	234 (58.1)	
N1	54 (9.5)	8 (4.8)	46 (11.4)	
N2	79 (13.9)	4 (2.4)	75 (18.6)	
N3	8 (1.4)	1 (0.6)	7 (1.7)	
NX	37 (6.5)	1 (0.6)	36 (8.9)	
Unknown	5 (0.9)	0 (0.0)	5 (1.2)	
Ferroptosis.scores (median [IQR])	1.47 [−5.53, 5.78]	2.61 [−1.80, 4.84]	−0.19 [−6.56, 7.11]	0.384
Group = High/Low (%)	314/254 (55.3/44.7)	115/50 (69.7/30.3)	199/204 (49.4/50.6)	<0.001

In order to dig deep into the relationship between ferroptosis genes and tumor characteristics, we explored all 382 ferroptosis genes reported in the FerrDb database, including 150 drivers, 123 markers, and 109 suppressors (Supplementary Tables S1–S4). Then we screened out the 234 validated ferroptosis genes (VFGs) from them to improve credibility. We used the ConsensusClusterPlus R package to classify patients with qualitatively different ferroptosis patterns based on the expression levels of 234 validated ferroptosis genes, and four distinct validated ferroptosis gene patterns were identified using unsupervised clustering (Figure 2D, Supplementary Figures S3A–H), including 244 cases in pattern A, 123 cases in pattern B, 93 cases in pattern C and 110 cases in pattern D. Then the cumulative distribution function (CDF) curve and scree plot were used to verify the rationality of the grouping (Supplementary Figure S3I, J). The track plot showed the details of grouping (Supplementary Figure S3K). We named these patterns as VFGcluster A-D, respectively. A dramatic difference was found on the FRG transcriptional profile among the four different VFG clusters (Figure 2E). VFGcluster A was characterized by decreased expression level of HSPA5, SLC7A11, NFE2L2, SAT1, NCOA4, MT1G, and DPP4, and presented variable increase in other FRGs; VFGcluster B exhibited a remarkable decrease in SLC7A11, RPL8, and GLS2 and an increase in NFE2L2, NCOA4, DPP4, ACSL4, and SAT1; VFGcluster C showed a significant increase in CDKN1A, HSPA5, MT1G, SAT1, DPP4, and ACSL4, and decrease in GLS2, ALOX15, and HSPB1; and VFGcluster D was characterized by increased expression of NFE2L2, HSPB1, SLC1A5, TFRC, NCOA4, and CS. We also noticed that VFGcluster B-C had higher TNM categories compared with VFGcluster A, but there existed no significant differences in gender and age among the four VFGclusters (Figure 2F). Survival analyses for the four VFGclusters revealed the particularly prominent survival advantage in patients from VFGcluster A (Figure 2G).

### TME Cell Infiltration Characteristics in Distinct VFG Patterns

**FIGURE 3:**
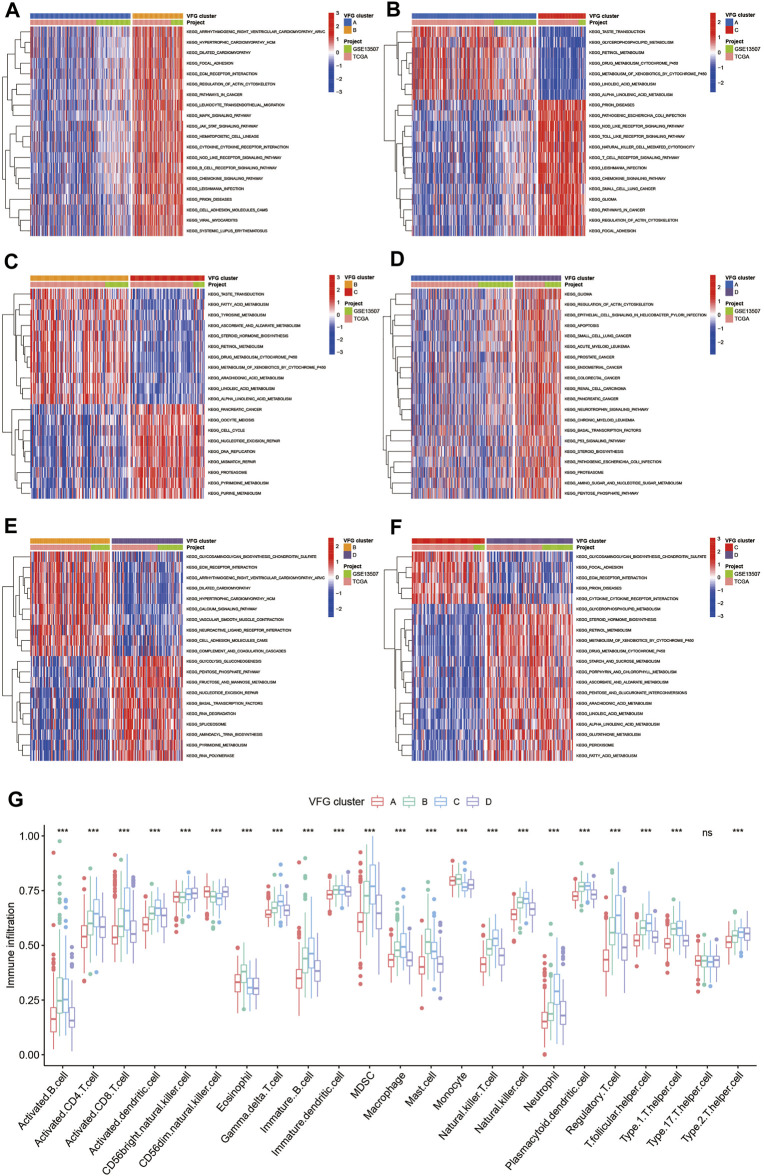
TME cell infiltration characteristics in distinct VFG patterns. **(A–F)** GSVA enrichment analysis showing the activation states of biological pathways in distinct ferroptosis patterns. The heatmap was used to visualize these biological processes, and red represented activated pathways and blue represented inhibited pathways. The project and VFGclusters were used as sample annotations. **(A)** VFGcluster A vs. VFGcluster B; **(B)** VFGcluster A vs. VFGcluster C; **(C)** VFGcluster B vs. VFGcluster C; **(D)** VFGcluster A vs. VFGcluster D; **(E)** VFGcluster B vs. VFGcluster D; **(F)** VFGcluster C vs. VFGcluster D. **(G)** The abundance of each TME infiltrating cell in four ferroptosis patterns. The upper and lower ends of the boxes represented interquartile range of values. The lines in the boxes represented median value, and black dots showed outliers. The asterisks represented the statistical *p* value (*p < 0.05; **p < 0.01; ***p < 0.001).

In order to further explore the latent differences in biological behaviors behind the distinct VFG patterns, we performed GSVA enrichment analysis. As shown in Figures 3A,B, VFGcluster A was dramatically enriched in pathways associated with metabolism, such as glycerophospholipid metabolism, linolenic metabolism, and drug metabolism mediated by cytochrome P450. VFGcluster B showed enrichment in stromal and carcinogenic activation pathways such as MAPK signaling pathway, focal adhesion, and ECM receptor interaction (Figure 3A), and it also exhibited relative enrichment in pathways associated with metabolism compared to VFGcluster C (Figure 3C). VFGcluster C was related to pathways about stroma, tumorigenesis, and infectious immunity (Figures 3B, 3C, 3F), while VFGcluster D was remarkably enriched in metabolic and carcinogenic activation pathways (Figures 3D–F). Then we analyzed the TME cell infiltration and were surprised to find that VFGcluster B and C were significantly enriched in nearly all kinds of immune cells such as activated CD4^+^ T cell, activated CD8^+^ T cell, activated dendritic cell, macrophage, MDSC, and natural killer cell (Figure 3G). However, patients with these VFG patterns did not show a corresponding survival advantage (Figure 2F). It has been reported that the innate immune cells as well as adaptive immune cells in TME could contribute to tumor progression (Hinshaw and Shevde, 2019). Previous studies have suggested that TME could be classified into three distinguished immune phenotypes based on the basic immune profiles: immune-inflamed phenotype, immune-excluded phenotype, and immune-desert phenotype. The immune-inflamed phenotype was characterized by abundant immune cells presented in the tumor parenchyma as well as many proinflammatory and effector cytokines. The immune-excluded phenotype was also abundant in various immune cells, however, the immune cells did not penetrate the tumor parenchyma and were retained in the stroma surrounding the tumor nests. The stroma could limit T-cell migration and their normal function of anti-tumor. However, the immune-desert phenotype was characterized by a lack of T cells in both the parenchyma and the stroma of the tumor (Chen and Mellman, 2017). The results of GSVA analyses have displayed that VFGcluster B and VFGcluster C were tightly connected with stroma activation. Therefore, we speculated that VFGclsuter B and VFGcluster C belonged to immune-excluded phenotype, in which the stroma activation significantly suppressed the immune cells’ normal anti-tumor function.

### Generation of Ferroptosis Gene Signatures and Functional Annotation

**FIGURE 4:**
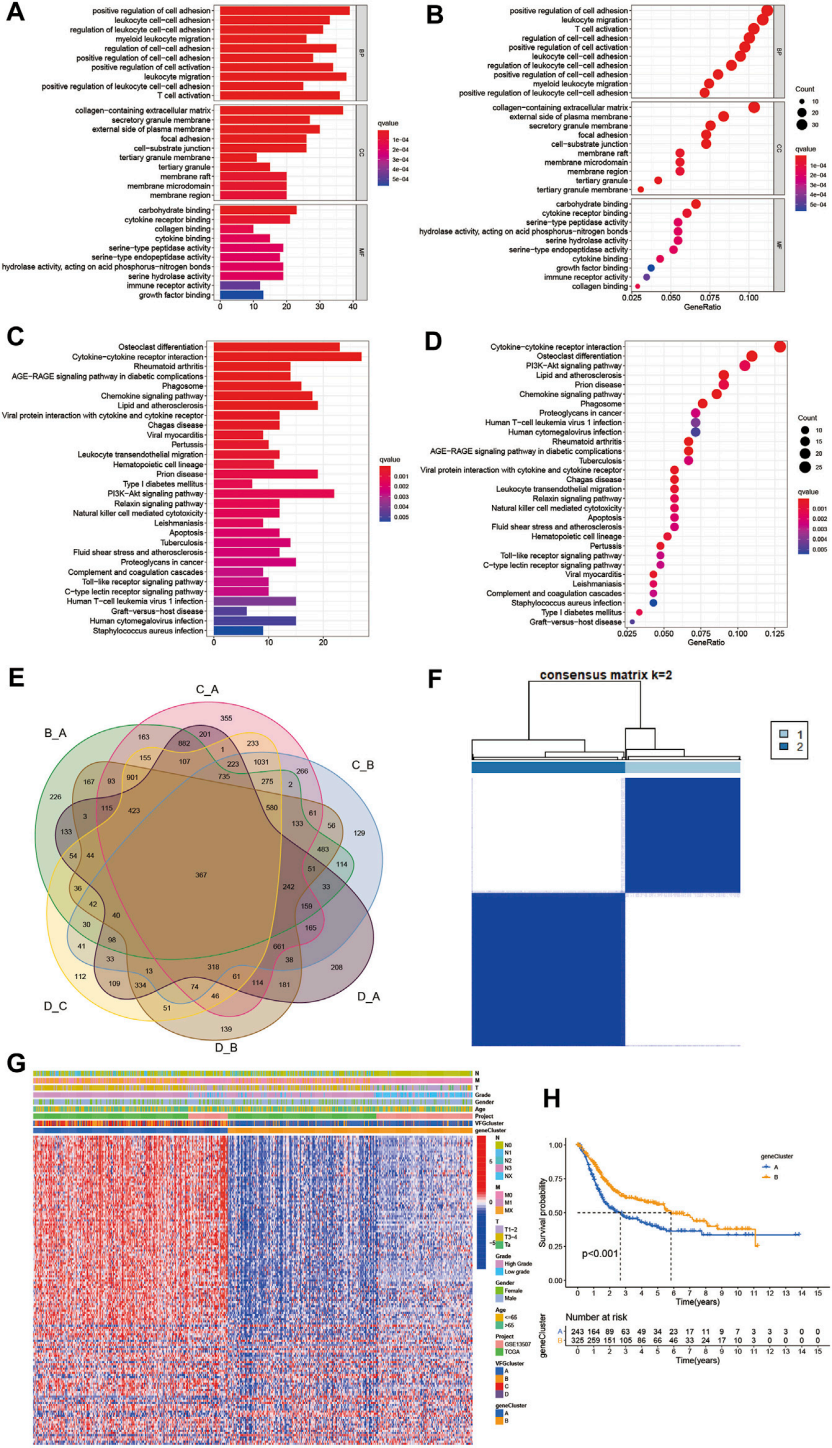
Generation of ferroptosis gene signatures and functional annotation. **(A,B)** Functional annotation for VFG cluster-related DEGs using GO enrichment analysis. The color depth of the barplots and plots represented the number of genes enriched. The pathways were grouped by cellular component (CC), molecular function (MF) and biological process (BP). **(C,D)** Functional annotation for VFG cluster-related DEGs using KEGG enrichment analysis. The color depth of the barplots and plots represented the number of genes enriched. **(E)** 367 VFG cluster-related DEGs shown in the Venn diagram. **(F)** Unsupervised clustering of 367 VFG cluster-related DEGs in meta-cohort and consensus matrices for *k* = 2. **(G)** Unsupervised clustering of overlapping 367 VFG cluster-related DEGs in meta-cohort to classify patients into different genomic subtypes, termed as gene cluster A-B, respectively. The gene clusters, VFGclusters, project, age, gender, grade, and TNM category were used as patient annotations. **(H)** Kaplan–Meier curves indicated ferroptosis genomic phenotypes were markedly related to overall survival of 568 patients in meta-cohort, of which 243 cases were in gene cluster A and 325 cases in gene cluster B (*p* < 0.001, Log-rank test).

**FIGURE 5:**
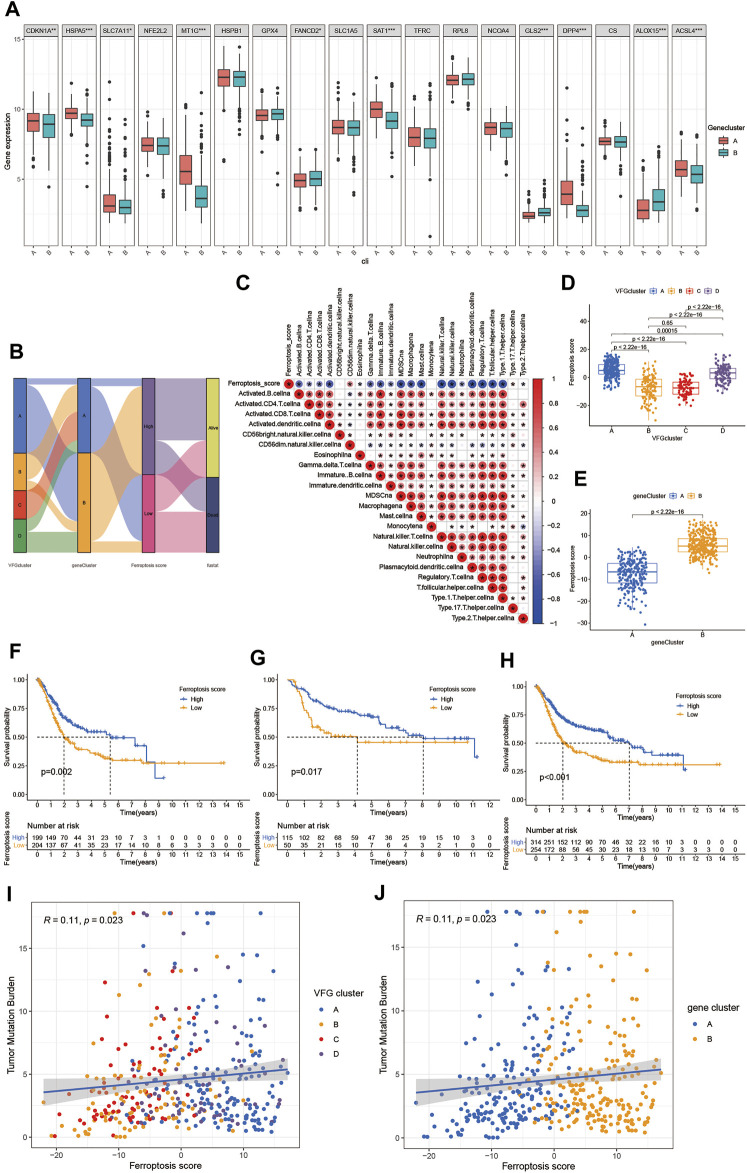
Establishment of the ferroptosis score and its interaction with tumor clinicopathological characteristics. **(A)** Expression of 18 FRGs in two gene clusters. The upper and lower ends of the boxes represented interquartile range of values. The lines in the boxes represented median value, and black dots showed outliers. The asterisks represented the statistical *p* value (*p < 0.05; **p < 0.01; ***p < 0.001). The student’s t test was used to test the statistical differences between two gene clusters. **(B)** Sankey diagram showing the changes of VFGclusters, survival status, gene cluster, and ferroptosis score. **(C)** Correlations between the ferroptosis score and the known immune cells in meta-cohort using Spearman analysis. Negative correlation was marked with blue and positive correlation with red. **(D)** Differences in the ferroptosis score among four VFGclusters in meta-cohort. The Kruskal Wallis H test was used to compare the statistical difference between four VFGclusters (*p* < 0.001). **(E)** Differences in the ferroptosis score among two gene clusters in meta-cohort (*p* < 0.001, Wilcoxon test). **(F)** Survival analyses for low (204 cases) and high (199 cases) ferroptosis score patient groups in the TCGA-BLCA cohort using Kaplan–Meier curves (*p* = 0.002, Log-rank test). **(G)** Survival analyses for low (50 cases) and high (115 cases) ferroptosis score patient groups in GSE13507 cohort using Kaplan–Meier curves (*p* = 0.017, Log-rank test). **(H)** Survival analyses for low (254 cases) and high (314 cases) ferroptosis score patient groups in meta-cohort using Kaplan–Meier curves (*p* < 0.001, Log-rank test). **(I)** Linear regression analysis for TMB and ferroptosis score. The dot represented each sample, and the color of the dot represented the VFGcluster. Blue, VFGcluster A; orange, VFGcluster B; red, VFGcluster C; purple, VFGcluster D (R = 0.11, *p* = 0.023). **(J)** Linear regression analysis for TMB and ferroptosis score. The dot represented each sample, and the color of the dot represented the gene cluster. Blue, gene cluster A; orange, gene cluster B (R = 0.11, *p* = 0.023).

To further investigate the latent biological behavior of each VFG pattern, we used limma R package to discover 367 VFG cluster-related DEGs (Figure 4E). We performed GO and KEGG enrichment analyses for the DEGs by using the clusterProfiler R package. To our surprise, the results of GO enrichment analysis showed a remarkable relationship with stroma and immunity in all cellular component (CC), molecular function (MF), and biological process (BP) patterns (Figures 4A,B). The genes in KEGG analysis also exhibited enrichment in pathways related to immunity, which was consistent with previous results (Figures 4C,D). The above results further proved that ferroptosis was an indispensable component in modification of immunity and TME. Then we performed unsupervised clustering analyses based on the 367 VFG cluster-related DEGs to find out the potential regulation mechanism. We successfully classified the patients into two distinct genomic subgroups using the unsupervised clustering algorithm (Figures 4F,G, Supplementary Figures S4A–H). The cumulative distribution function (CDF) curve and screen plot also validated the rationality of the grouping (Supplementary Figures S4I–K). We named the two different subgroups as gene cluster A and B, respectively. In total, 243 patients were assigned to gene cluster A while 325 patients were classified into gene cluster B. We observed that gene cluster B was mainly composed of patients from VFGcluster A, and tumors in gene cluster B had a better TNM category and were enriched in low grade compared to gene cluster A (Figure 4G). Thus, it is not difficult to explain the phenomenon that patients in gene cluster B had a better prognosis (Figure 4H). We also found that the two gene clusters were characterized by different signature genes (Figure 4G). We also discovered a significant difference in expression level among the majority of FRGs, which was consistent with VFGclusters (Figure 5A).

### Characteristics of Clinical Traits in Ferroptosis Related Phenotypes

**FIGURE 6:**
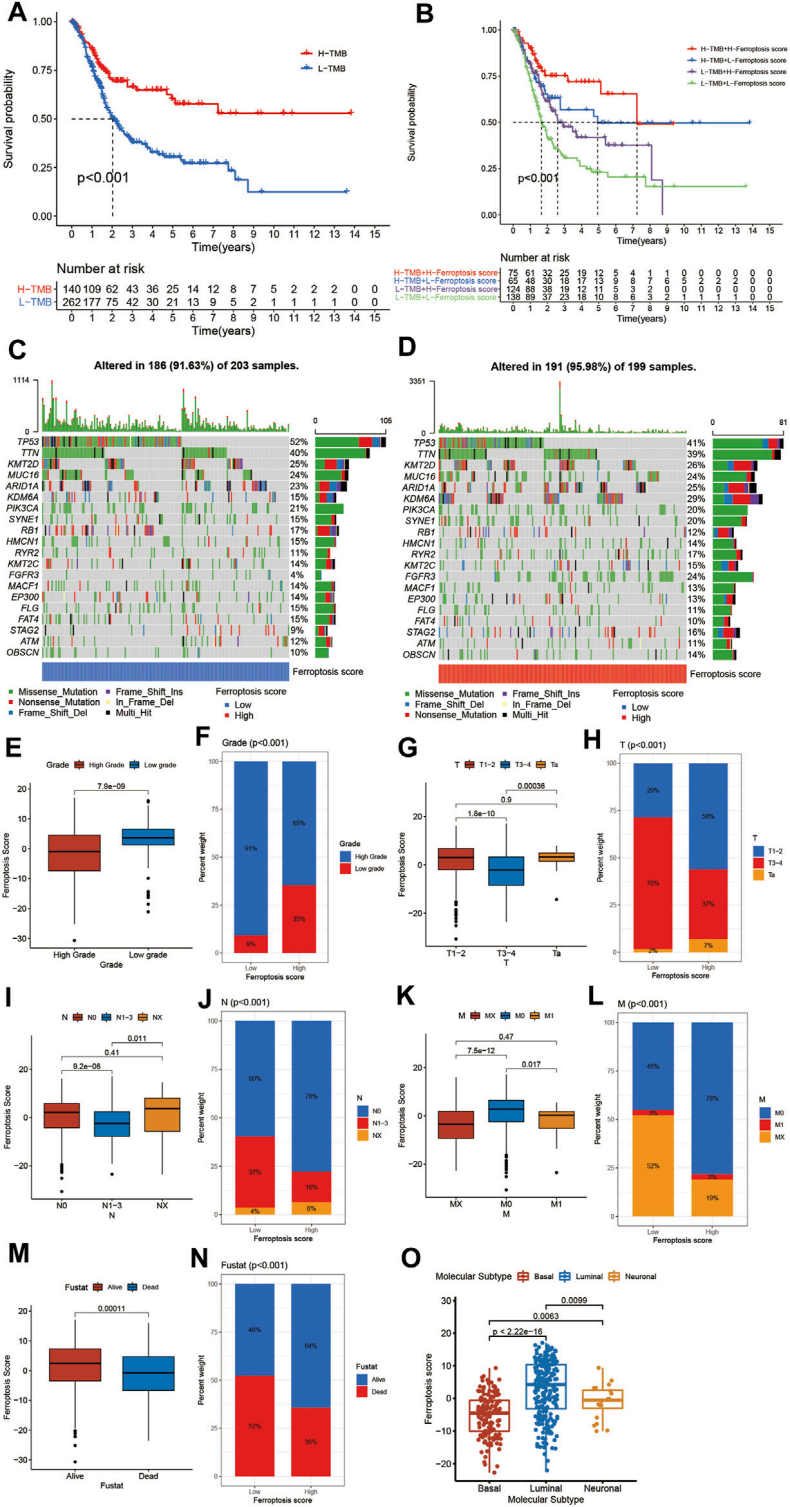
Characteristics of ferroptosis in tumor somatic mutation and tumor stage. **(A)** Survival analyses for low (262 cases) and high (140 cases) TMB patient groups in the TCGA-BLCA cohort using Kaplan–Meier curves (*p* < 0.001, Log-rank test). **(B)** Survival analyses for four groups grouped according to TMB and ferroptosis score in the TCGA-BLCA cohort using Kaplan–Meier curves including 75 cases in the high TMB and high ferroptosis score group, 65 cases in the high TMB and low ferroptosis score groups, 124 cases in the low TMB and high ferroptosis score group, and 138 cases in the low TMB and low ferroptosis score group. The high TMB and high ferroptosis score group showed significantly better overall survival than the other three groups (*p* < 0.001, Log-rank test). **(C,D)** Waterfall plot of tumor somatic mutation established by those with low ferroptosis score **(C)** and high ferroptosis score **(D)**. Each column represented individual patients. The upper barplot showed TMB. The number on the right indicated the mutation frequency in each gene. The right barplot showed the proportion of each variant type. **(E)** Differences in the ferroptosis score between high and low tumor grade groups in meta-cohort (*p* < 0.001, Wilcoxon test). **(F)** Proportion of patients with different tumor grade in low or high ferroptosis score groups. High grade/low grade: 91%/9% in the low ferroptosis score groups and 65%/35% in the high ferroptosis score groups. **(G)** Differences in the ferroptosis score among Ta, T1-T2, and T3-T4 groups in meta-cohort (*p* < 0.001, Kruskal Wallis H test). **(H)** Proportion of patients with Ta, T1-T2, and T3-T4 stage tumor in the low or high ferroptosis score groups. **(I)** Differences in the ferroptosis score among N0, N1-N3, and NX groups in meta-cohort (*p* < 0.001, Kruskal Wallis H test). **(J)** Proportion of patients with N0, N1-N3, and NX stage tumor in low or high ferroptosis score groups. **(K)** Differences in the ferroptosis score among M0, M1, and MX groups in meta-cohort (*p* < 0.001, Kruskal Wallis H test). **(L)** Proportion of patients with M0, M1, and MX stage tumor in the low or high ferroptosis score groups. **(M)** Differences in the ferroptosis score between alive and dead groups in meta-cohort (*p* < 0.001, Wilcoxon test). **(N)** Proportion of alive patients in the low or high ferroptosis score groups. **(O)** Differences in the ferroptosis score among three subtypes of BCa including basal, luminal and neuronal subtypes (Kruskal Wallis H test).

The above analyses revealed a remarkable correlation between TME and ferroptosis based on the patient population. Therefore, we next explored the latent ferroptosis pattern in individual patients considering the individual heterogeneity and complexity of ferroptosis. Based on these phenotype-related genes, we constructed a set of scoring system named the ferroptosis score to quantify the ferroptosis pattern of individual patients with BCa. The Sankey diagram was used to visualize the attribute changes of individual patients (Figure 5B). To better understand the relationship between ferroptosis signature and TME, we also tested the correlation between the known immune cells and ferroptosis score (Figure 5C). We found significant difference in the ferroptosis score between VFGclusters using the Kruskal Wallis H test (Figure 5D). VFGcluster A showed the highest median score while VFGcluster B and C shared the lowest median score, which indicated that the low ferroptosis score might be related to stroma activation signatures. Moreover, gene cluster B also exhibited a higher median ferroptosis score compared to gene cluster A and the difference was of statistical significance (Figure 5E). Next, we further explored whether the ferroptosis score had a predictive significance for the prognosis of patients. We used survminer R package to determine the cut off value -0.041 and divided the patients into two subgroups with high and low ferroptosis score. We found a significant survival advantage among patients with high ferroptosis score in all of the GSE13507 cohort (Figure 5G), TCGA-BLCA cohort (Figure 5F), and the meta-cohort (Figure 5H). In the meta-cohort, the 5 year survival rate with high ferroptosis score is almost twice than those with low ferroptosis score (22.29% vs. 11.81%). Then, we investigated the interaction between the ferroptosis score and clinical signatures and found the ferroptosis score was significantly related to the grade, TNM category, and final survival status (Figures 6E–6N). We also found significant differences of the ferroptosis score in the molecular subtypes of BCa (Figure 6O). However, the distribution difference of the ferroptosis score in age and gender did not show a statistical significance (Supplementary Figures S5A–D). In addition, we performed subgroup analyses and found the ferroptosis score was a good predictor of survival especially for patients who were male, with high grade and low TNM category (T1-T2, N0-N3, M0) (Supplementary Figures S5E–S).

### Characteristics of Ferroptosis in Tumor Somatic Mutation, Immunotherapy and Chemotherapy

Next, we sought to explore the relationship between the ferroptosis score and TMB. We discovered that TMB was positively correlated to the ferroptosis score. Compared to other clusters, VFGcluster A had a higher ferroptosis score, so did gene cluster B, which was consistent to above results (Figures 5I,J). Then, we analyzed the distribution differences of somatic mutation between the low and high ferroptosis scores in the TCGA-BLCA cohort using maftools R package. As shown in Figures 6C,D, in general, there were no obvious distribution differences of TMB between the low and high ferroptosis scores, but for some popular genes in BCa studies such as FGFR3, the high ferroptosis score group exhibited more extensive TMB than the low ferroptosis score group. The previous studies have demonstrated that TMB was tightly related to the results of immunotherapy and the prognosis of patients (Chan et al., 2019; Valero et al., 2021). Therefore, we first preformed survival analyses to validate the linkage between TMB and clinical outcome and were excited to find that patients with high ferroptosis score and high TMB had a better prognosis, which indicated the combination of ferroptosis score and TMB had a considerable prognostic value for BCa patients (Figures 6A,B).

**FIGURE 7:**
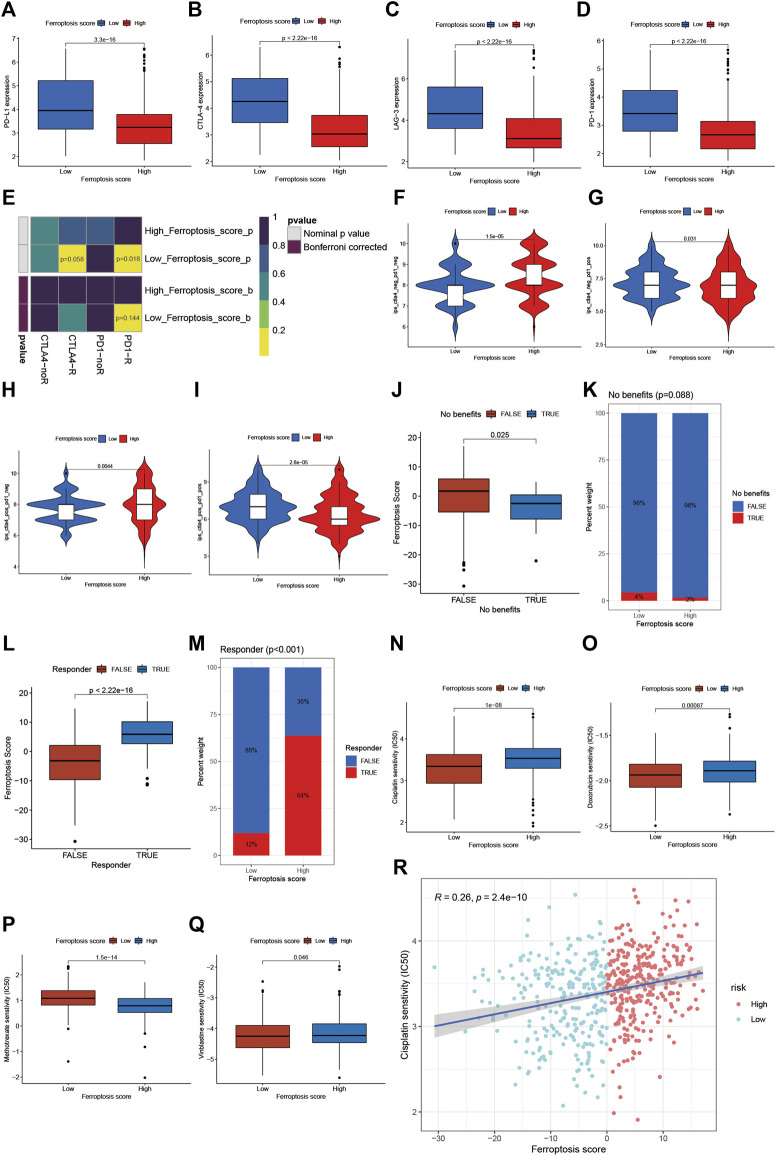
Role of ferroptosis patterns in immunotherapy and chemotherapy. **(A)** Differences in the expression of PD-L1 between the high and low ferroptosis score groups in meta-cohort (*p* < 0.001, Wilcoxon test). **(B)** Differences in the expression of CTLA-4 between high and low ferroptosis score groups in meta-cohort (*p* < 0.001, Wilcoxon test). **(C)** Differences in the expression of LAG-3 between high and low ferroptosis score groups in meta-cohort (*p* < 0.001, Wilcoxon test). **(D)** Differences in the expression of PD-1 between high and low ferroptosis score groups in meta-cohort (*p* < 0.001, Wilcoxon test). **(E)** The similarity of gene expression profiles between ferroptosis score and BCa patients treated with immune checkpoint blockade (ICB). CTLA4-noR, patients no respond to anti-CTLA4 treatment, CTLA4-R, patients respond to anti-CTLA4 treatment, PD1-noR, patients no respond to anti-PD-1 treatment, PD1-R, patients respond to anti-PD-1 treatment. **(F–I)** Violin diagram showed the differences of response index between high and low ferroptosis score groups in four subgroups. **(F)** If no immunotherapy was conducted, the high ferroptosis score resulted in a better prognosis compared to low ferroptosis score (*p* < 0.001, Wilcoxon test). **(G)** If only anti-PD1 immunotherapy was used, the high ferroptosis score resulted in a worse prognosis compared to low ferroptosis score (*p* = 0.031, Wilcoxon test). **(H)** If only anti-CTLA4 immunotherapy was used, the higher ferroptosis score group tended to get a better therapeutic response compared to low ferroptosis score group (*p* = 0.0044, Wilcoxon test). **(I)** When anti-PD1 and anti-CTLA4 immunotherapy methods were simultaneously adopted, the high ferroptosis score group might get significantly worse prognosis compared to low ferroptosis score group (*p* < 0.001, Wilcoxon test). **(J)** Differences in ferroptosis score between immunotherapy benefit and no benefit groups in meta-cohort (*p* = 0.025, Wilcoxon test). **(K)** The proportion of patients benefit from immunotherapy in low or high ferroptosis score groups. **(L)** Differences in ferroptosis score between immunotherapy response and nonresponse groups in meta-cohort (*p* < 0.001, Wilcoxon test). **(M)** The proportion of patients who response to immunotherapy in low or high ferroptosis score groups. **(N–Q)** Differences in IC50 of chemotherapy drugs between high and low ferroptosis score groups in meta-cohort. **N** cisplatin (*p* < 0.001, Wilcoxon test). **(O)** doxorubicin (*p* < 0.001, Wilcoxon test). **(P)** methotrexate (*p* < 0.001, Wilcoxon test). **(Q)** vinblastine (*p* = 0.046, Wilcoxon test). **(R)** Linear regression analysis for cisplatin sensitivity and ferroptosis score. The dot represented each sample, and the color of the dot represented the level of risk. Blue, low risk; red, high risk (R = 0.26, *p* < 0.001).

Then, we further explored whether the ferroptosis score had a predictive significance for the outcome of immunotherapy. As shown in Figures 7A–D, the expression of the main immunotherapy targets PD-1, PD-L1, LAG-3, and CTLA-4 were significantly lower in patients with high ferroptosis score compared to those with low ferroptosis score. Next, we evaluated the interaction between the ferroptosis score and the response to immune checkpoint inhibitors treatment. We found that the low ferroptosis score was related to a better response to anti-PD-1 and anti-CTLA-4 immunotherapy. After Bonferroni correction, there still existed a remarkable correlation between the low ferroptosis score and response to anti-PD-1 immunotherapy although there was a lack of statistical significance (Figure 7E). Then we divided the patients into four subgroups according to the use of anti-CTLA-4 and anti-PD-1 immunotherapy: CTLA-4 positive PD-1 positive, CTLA-4 positive PD-1 negative, CTLA-4 negative PD-1 positive, and CTLA-4 negative PD-1 negative. As shown in Figures 7F–I, in CTLA-4 positive PD-1 negative and CTLA-4 negative PD-1 negative subgroups, the high ferroptosis score was related to a better immunotherapy response, while in CTLA-4 positive PD-1 positive and CTLA-4 negative PD-1 positive subgroups the results were exactly opposite, which further proved that ferroptosis had a tighter relationship with immunotherapy targeted at PD-1 compared to other immune checkpoint inhibitors. Anti-PD-L1 immunotherapy has also been proven effective for patients with metastatic urothelial carcinoma in a multicenter, single-arm phase 2 trial using atezolizumab (IMvigor 210, NCT02108652) (Rosenberg et al., 2016). Using the data acquired from IMvigor 210 cohort, we further verified the interaction between the ferroptosis score and immune phenotypes. We found there existed significant differences in the proportion of three immune phenotypes between low and high ferroptosis groups (Supplementary Figure S6A). The immune-desert phenotype exhibited the highest ferroptosis score, whereas the immune-inflamed phenotype showed the lowest ferroptosis score (Supplementary Figures S6B), which was in accordance with the previous results. In general, a lower ferroptosis score predicted a better immunotherapy response, and vice versa (Figure 7J–M). Next, we would like to find out whether the ferroptosis score was also connected with response to chemotherapy. We screened out several commonly used chemotherapy drugs in BCa and explored the interaction between the half maximal inhibitory concentration (IC50) and ferroptosis score. We found that the low ferroptosis score was related to low IC50 in cisplatin, doxorubicin, and vinblastine (Figures 7N, O, Q), which means a higher sensitivity to chemotherapy. While methotrexate was just the reverse (Figure 7P). Then we further discovered that IC50 for cisplatin was positively correlated to the ferroptosis score (Figure 7R). In summary, the above results showed the unique role of the ferroptosis score in predicting the efficacy of immunotherapy and chemotherapy.

## Discussion

Nowadays, increasing evidences have demonstrated that ferroptosis could play a vital role in cancer therapy and predicting the prognosis of patients with cancer (Mou et al., 2019), including BCa. For example, Yan et al. has established a prognostic signature based on 6 ferroptosis regulator genes which could not only predict the progression of BCa patients but also the landscape of macrophage infiltration and EMT status (Yan et al., 2021). Moreover, a ferroptosis-related long non-coding RNA (FRlncRNA) signature comprising 13 prognostic FRlncRNAs established by Cui and his colleagues also had an independent prognostic significance for the overall survival of BCa patients. However, previous studies paid more attention to limited ferroptosis regulator genes and did not go deep into the comprehensive effect of ferroptosis in BCa as well as interaction between TME cell infiltration and ferroptosis, which were necessary to guide more effective immunotherapy strategies or therapies targeted at ferroptosis.

In this article, we first summarized the landscape of genetic variation of 23 FRGs in BCa among 412 samples from the TCGA-BLCA cohort, then we explored the FerrDb database to collect all 382 ferroptosis genes ever reported, screened out 234 validated ferroptosis genes, combined the GEO dataset GSE13507 and TGCA-BLCA cohort into a new meta-cohort, and divided the patients in the meta-cohort into four ferroptosis patterns named VFGcluster A-D using unsupervised clustering according to the expression levels of these validated ferroptosis genes. To our surprise, we not only found significant differences in clinical characteristics and the prognosis of patients among the four distinct VFG clusters, but also found remarkable differences in TME immune cell infiltration. TME comprises both cancer cells and immune cells including T cells, B cells, natural killer cells (NK cells), macrophages, dendritic cells (DCs), and myeloid-derived suppressor cells (MDSCs) (Binnewies et al., 2018). It was intriguing that VFGcluster B and C were abundant in almost all kinds of immune cells but did not exhibit consistent survival advantage. Therefore we classified the VFGcluster B and C into immune-excluded phenotype, also called ‘cold’ tumor, in which the majority of cytotoxic T lymphocytes (CTLs) were arrested in the margin of the tumor mass instead of the core region, thus having poor response to immunotherapy (Binnewies et al., 2018). Although both the immune cells and cancer cells in the TME share similar growth signals and metabolic properties, there still exist differences in the sensitivity to ferroptosis among these cells. For example, it seems that anti-tumor T cells are sensitive to ferroptosis while MDSCs exhibit resistance to ferroptosis, and M1 macrophages show higher resistance to ferroptosis than M2 phenotypes (Xu et al., 2021). Therefore, it is conceivable that the TME immune cell infiltration is tightly related to the VFG patterns. We also performed GSVA enrichment analysis and found the pathways related to tumorigenesis and stroma activation were remarkably enriched in VFGcluster B and C. Various studies have demonstrated that stroma could prevent CTL from entering the tumor core and suppress their anti-tumor function. Cells in the stroma such as fibroblasts could not only synthesize and secret collagen to form mechanical separation, but also secretes signaling molecular like transforming growth factor β (TGF-β), which was proved immunosuppressive. The combination of TGF-β blocking antibody and anti-PD-L1 reduced TGF-β signaling in stromal cells, facilitated T-cell penetration into the center of the tumor, and significantly restored anti-tumor immunity and suppressed tumor progression (Mariathasan et al., 2018). Therefore, the results of GSVA enrichment analysis were consistent with VFGcluster patterns.

Further, in this study, we explored the mRNA transcriptome differences between distinct VFG patterns and also found a remarkable relationship with stroma and immunity-related pathways. These differentially expressed genes were considered as ferroptosis-related signature genes. Then we classified the patients into two distinct genomic subtypes based on the 367 VFG cluster-related DEGs and found the gene clusters were tightly connected with VFGcluster patterns. These results demonstrated again that ferroptosis was an important signature to distinguish different TME landscapes. Therefore, a comprehensive assessment of the ferroptosis patterns will enhance our understanding of TME cell-infiltrating characterization.

Next, considering the individual heterogeneity and complexity of ferroptosis, it was necessary for us to explore the latent ferroptosis pattern in individual patients. Thus, we constructed a set of scoring system named the ferroptosis score to quantify the ferroptosis pattern of individuals with BCa. We found immune cells in TME were significantly related to the ferroptosis score and there also existed differences in the ferroptosis score among distinct VFG clusters. VFGcluster A showed the highest median score while VFGcluster B and C shared the lowest median score, which suggested the ferroptosis score was a reliable and effective tool to assess the individual ferroptosis patterns and could also be used to evaluate the landscape of TME immune cell infiltration. Moreover, we also discovered that the ferroptosis score was tightly interacted with clinical signatures such as the TNM category and tumor grade and could predict the prognosis of patients with BCa, especially for patients who were male, with high grade and low TNM category.

Our study also found that TMB was positively correlated to the ferroptosis score. The previous studies have reported that TMB could serve as a latent biomarker of the response to immunotherapy using checkpoint inhibitors in multiple cancers such as lung cancer and mesothelioma (Harber et al., 2021; Sholl, 2021). Therefore, we would like to figure out whether the ferroptosis score could predict the response to immunotherapy and guide clinical treatment strategies. Many patients have benefited from immunotherapy using immune checkpoint inhibitors such as PD-1, PD-L1, and CTLA-4 blockade, but many more patients did not see pronounced clinical response to immunotherapeutic intervention (Binnewies et al., 2018). PD-1/PD-L1 blockade has demonstrated a significant benefit in patients with unresectable and metastatic BCa in the second-line setting, either as monotherapy or in combination with chemotherapy or CTLA-4 checkpoint inhibition (Witjes et al., 2021). The results of the phase II trial using the PD-1 inhibitor pembrolizumab reported a complete pathological remission (pT0) in 42% and pathological response (<pT2) in 54% of patients (Necchi et al., 2018), whereas another single-arm phase II trial with atezolizumab showed a pathologic complete response rate of 31% (Powles et al., 2019). These results suggested that the response rate still needed to be improved and it was important to screen out patients who were appropriate for immunotherapy. Our results found that the lower ferroptosis score was connected with higher expression of main immunotherapy targets like PD-1, PD-L1, LAG-3, and CTLA-4 and a better response to immunotherapy using PD-1 blockade. Therefore, we showed that ferroptosis patterns played a non-negligible role in distinguishing different TME and ferroptosis signature integrated with various biomarkers comprising TMB, immune checkpoint expression, landscape of TME immune cell infiltration and stromal activation, and could be an effective predictive strategy for immunotherapy.

Many drugs used for cancer treatment have been confirmed to work as ferroptosis inducer in their anti-tumor function, such as cisplatin and sorafenib (Liang et al., 2019). Therefore, in our study, we evaluated the relationship between the ferroptosis score and sensitivity to different chemotherapy drugs and found IC50 for all these drugs exhibited a significant difference between the high and low ferroptosis score groups, which indicated that the ferroptosis score could also be a feasible indicator for the response to chemotherapy. Since cisplatin-based chemotherapy was a conventional treatment for patients with BCa (Sylvester et al., 2021; Witjes et al., 2021), we further performed regression analysis for the ferroptosis score and IC50 for cisplatin and confirmed that there really existed a positive correlation between the sensitivity to cisplatin chemotherapy and ferroptosis score. Overall, the ferroptosis score could also be an effective predictive strategy for chemotherapy, which could help in selecting drug resistant patients before treatment.

In general, our study provided a comprehensive insight into the interaction between ferroptosis, TMB, TME immune cell infiltration, chemotherapy, and immunotherapy. We demonstrated that different VFG patterns could help in distinguishing the landscape of TME immune cell infiltration and clinical characteristics among patients, which was further verified using the ferroptosis score within individuals. We also demonstrated that the ferroptosis score could be used to evaluate the clinicopathological features including the TNM category, tumor grade, TMB, and genetic variation. Moreover, the ferroptosis score could also function as a predictive indicator for the survival of patients. Finally, we also evaluated the ability of the ferroptosis score to predict the response to immunotherapy using immune checkpoint inhibitor and chemotherapy, which might help in improving therapeutic strategies, screening patients eligible for immunotherapy or chemotherapy and guiding individual precision therapy in the future.

However, we also realize that there still exist several shortcomings and limitations in our study. First, the current omics data only provide the level of mRNA but the ferroptosis process relies on proteins, which will bring in some inaccuracies. Second, although we have used the data acquired from IMvigor 210 cohort to further verify the role of ferroptosis patterns in immunotherapy, the number of clinical samples is limited and our study is a lack of verification from other clinical data sets apart from the public data which will be helpful to further confirm our conclusions, and whether ferroptosis has a similar role in other types of cancer hasn’t been verified. Therefore, we are prepared to collect some clinical samples to further verify our conclusions, and assess the role of ferroptosis in other urinary system tumors. Third, since some new studies were published and novel ferroptosis-related genes were reported recently, the ferroptosis-related genes we used for analyses could not be comprehensive enough, which might bring a bias into our study. Finally, the specific mechanisms behind the interaction between ferroptosis patterns and TMB immune cell infiltration remain unclear, so cell biological experiments should be performed for further validation in the future.

In conclusion, our work demonstrated and interpreted the complicated regulation mechanisms of ferroptosis on the tumor microenvironment. The differences in ferroptosis patterns in population or individual patients could significantly influence the heterogeneity in tumor clinicopathological features and TME, thus influencing the response to immunotherapy and chemotherapy. Therefore, better understanding and evaluating ferroptosis patterns could be helpful in guiding the clinical therapeutic strategy and improving the prognosis of patients with BCa.

## Data Availability

The original contributions presented in the study are included in the article/Supplementary Material, further inquiries can be directed to the corresponding author.
