# Supplementary figures and images for: Ferroptosis Patterns and Tumor Microenvironment Infiltration Characterization in Bladder Cancer
Source: Front Cell Dev Biol. 2022 Mar 21;10:832892. doi: 10.3389/fcell.2022.832892 (PMC8978677; doi:10.3389/fcell.2022.832892)

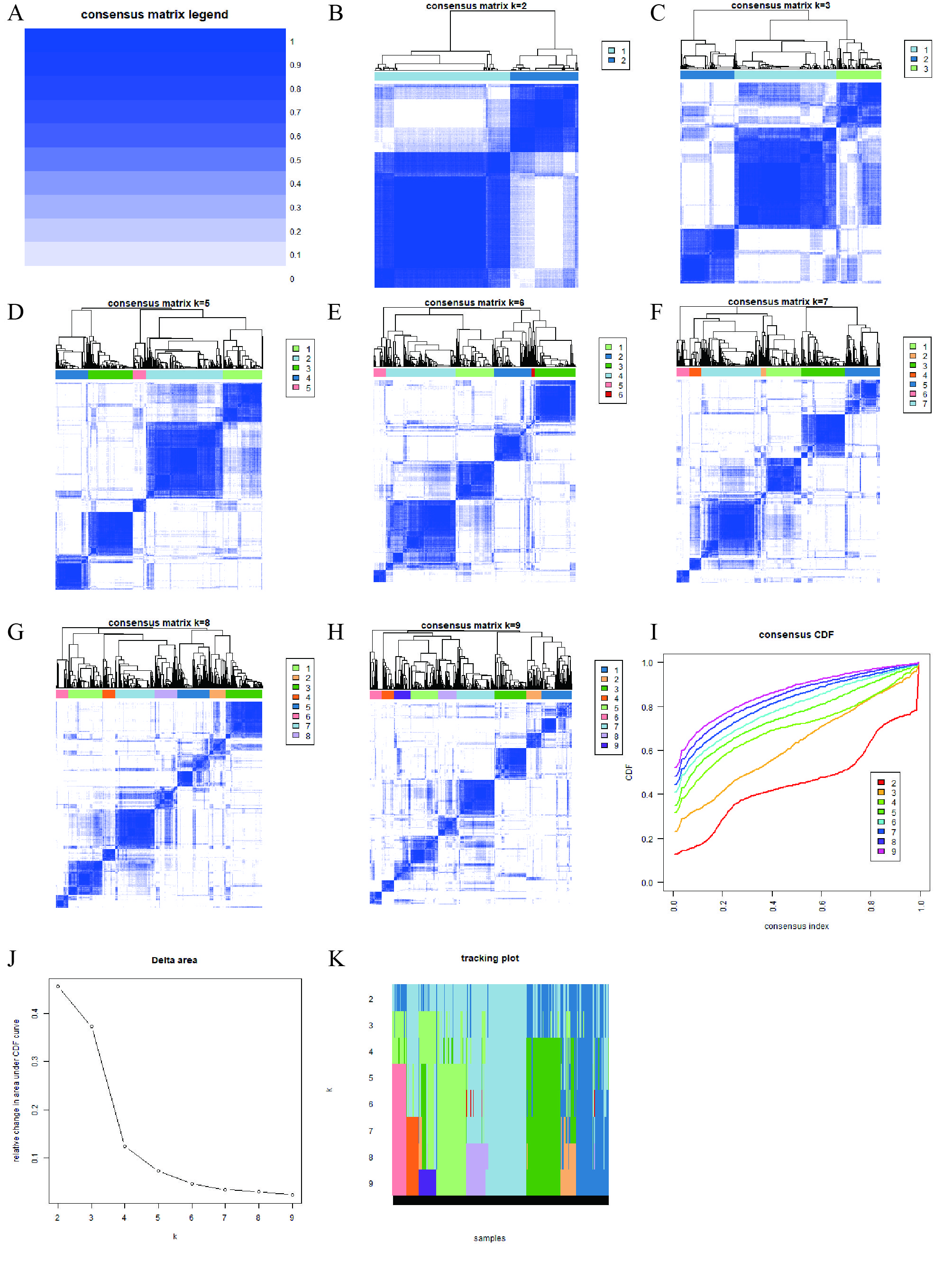

Supplement: Supplementary file 2 [file Image3.JPEG]

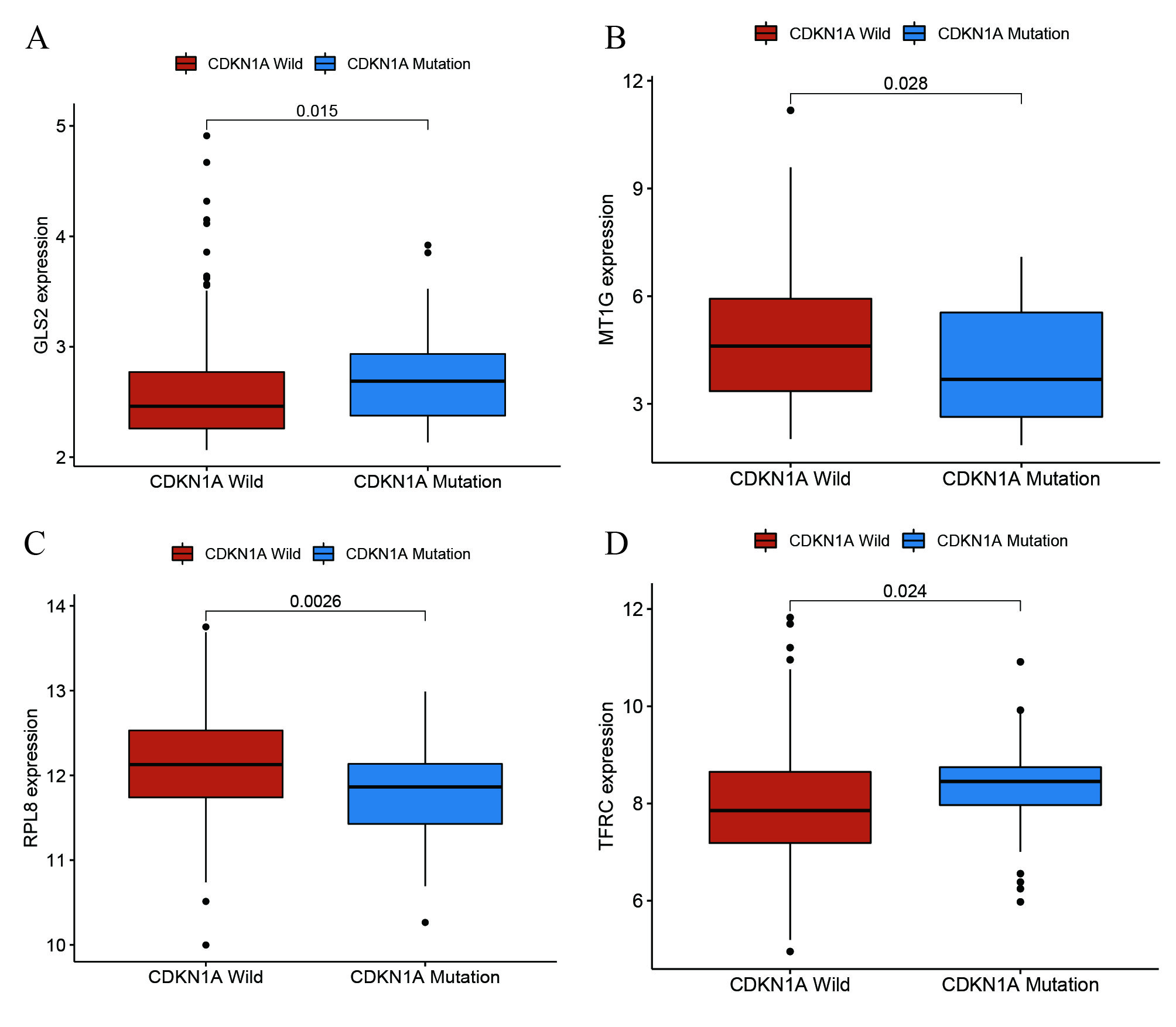

Supplement: Supplementary file 4 [file Image1.JPEG]

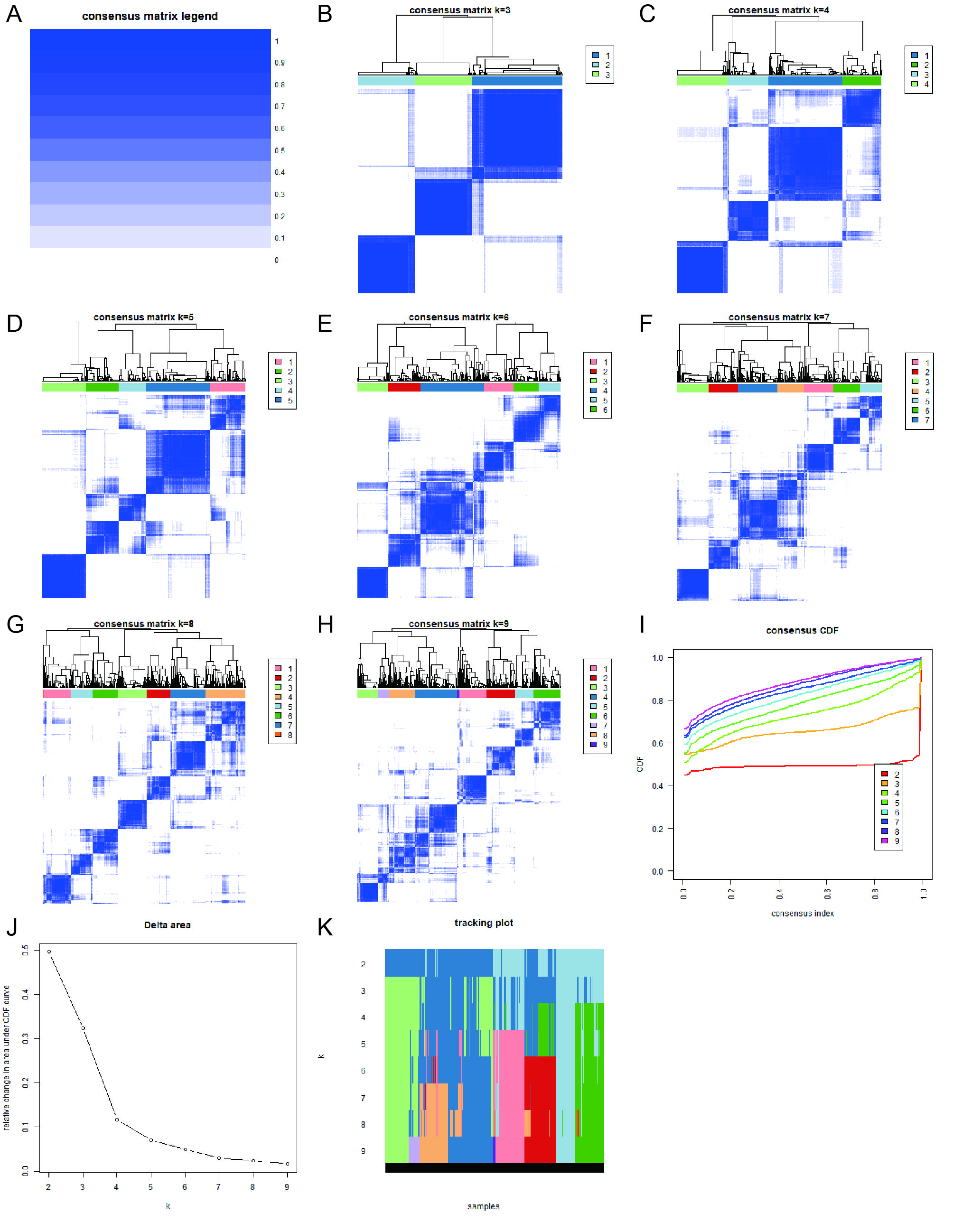

Supplement: Supplementary file 5 [file Image4.JPEG]

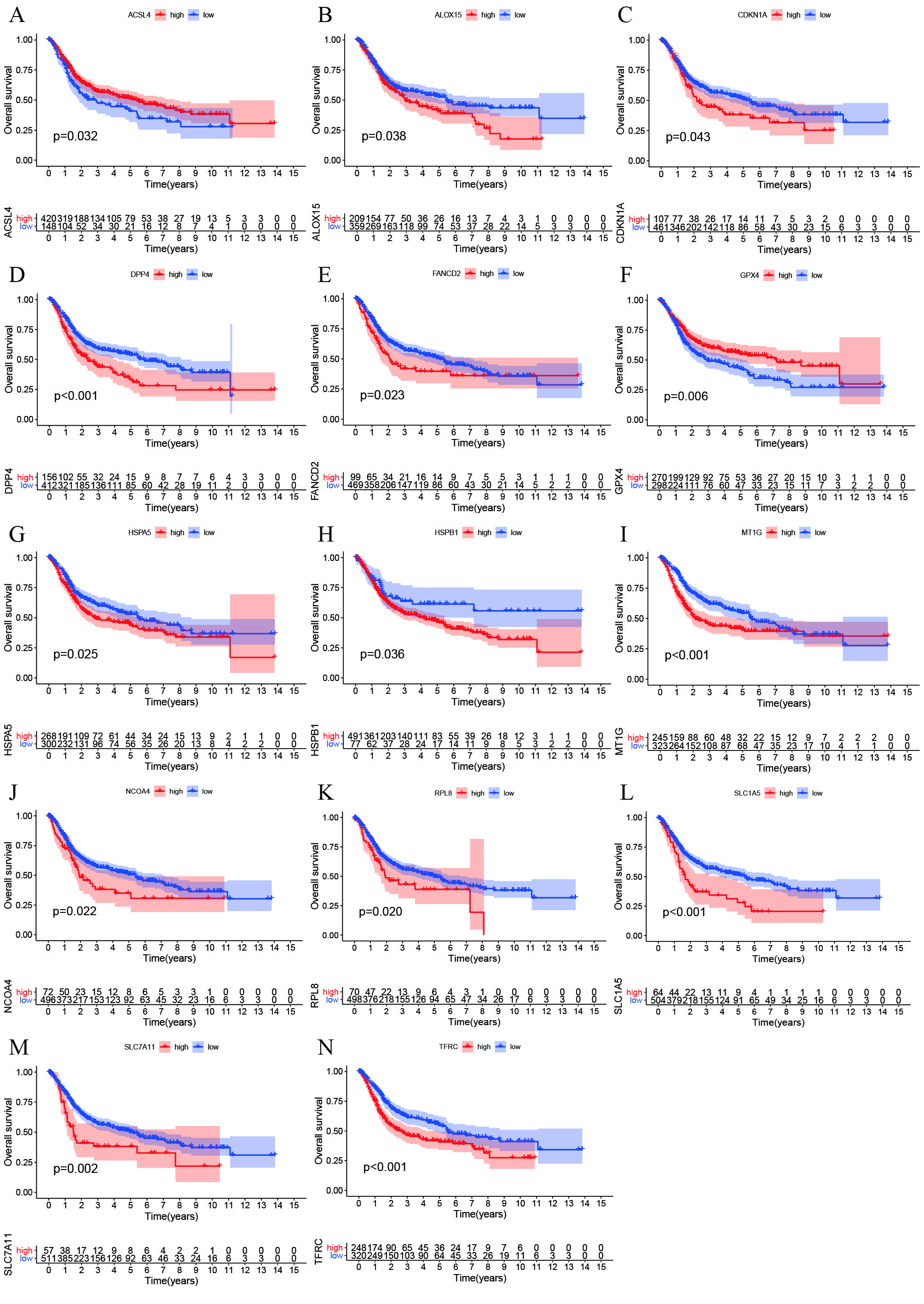

Supplement: Supplementary file 6 [file Image2.JPEG]

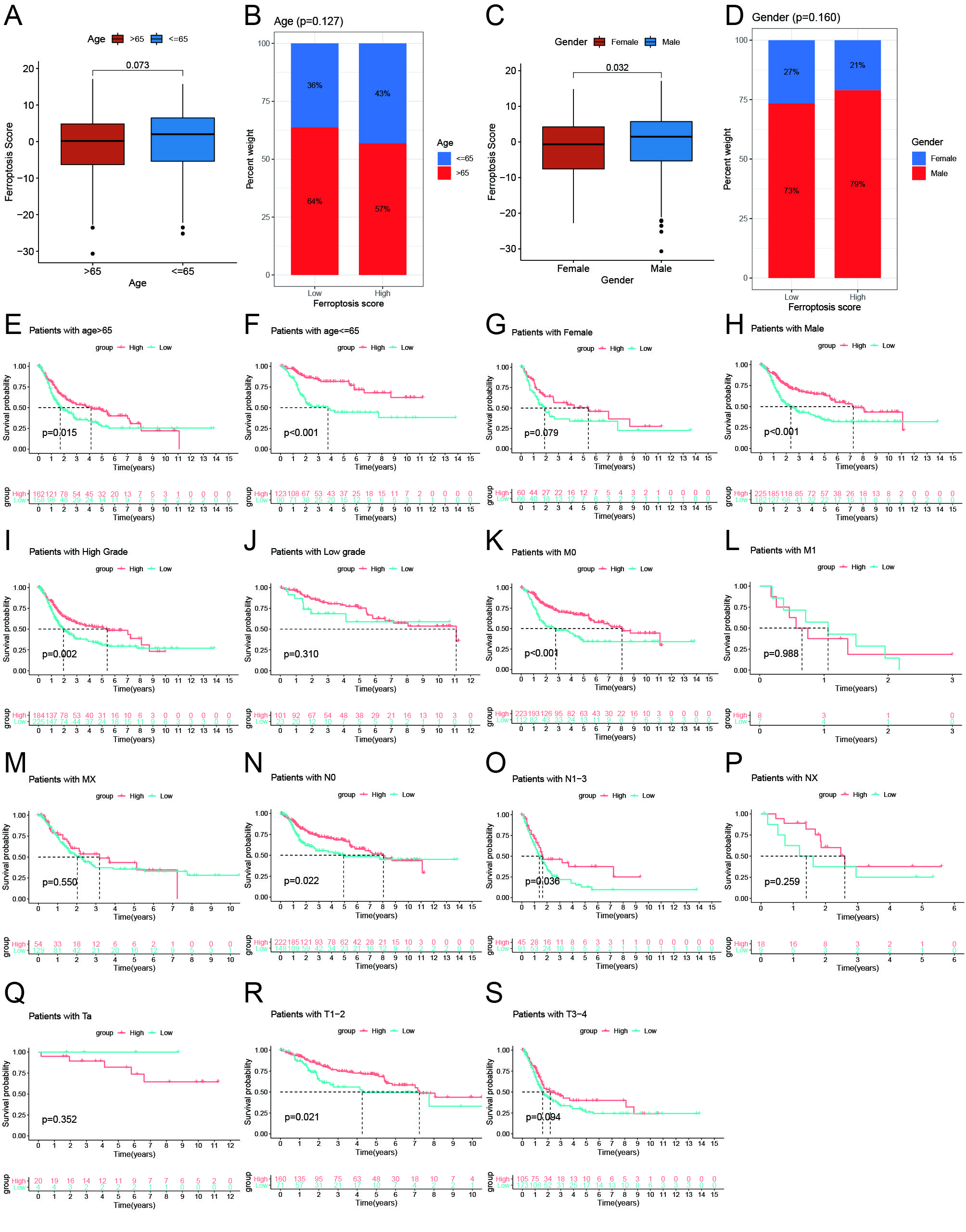

Supplement: Supplementary file 7 [file Image5.JPEG]

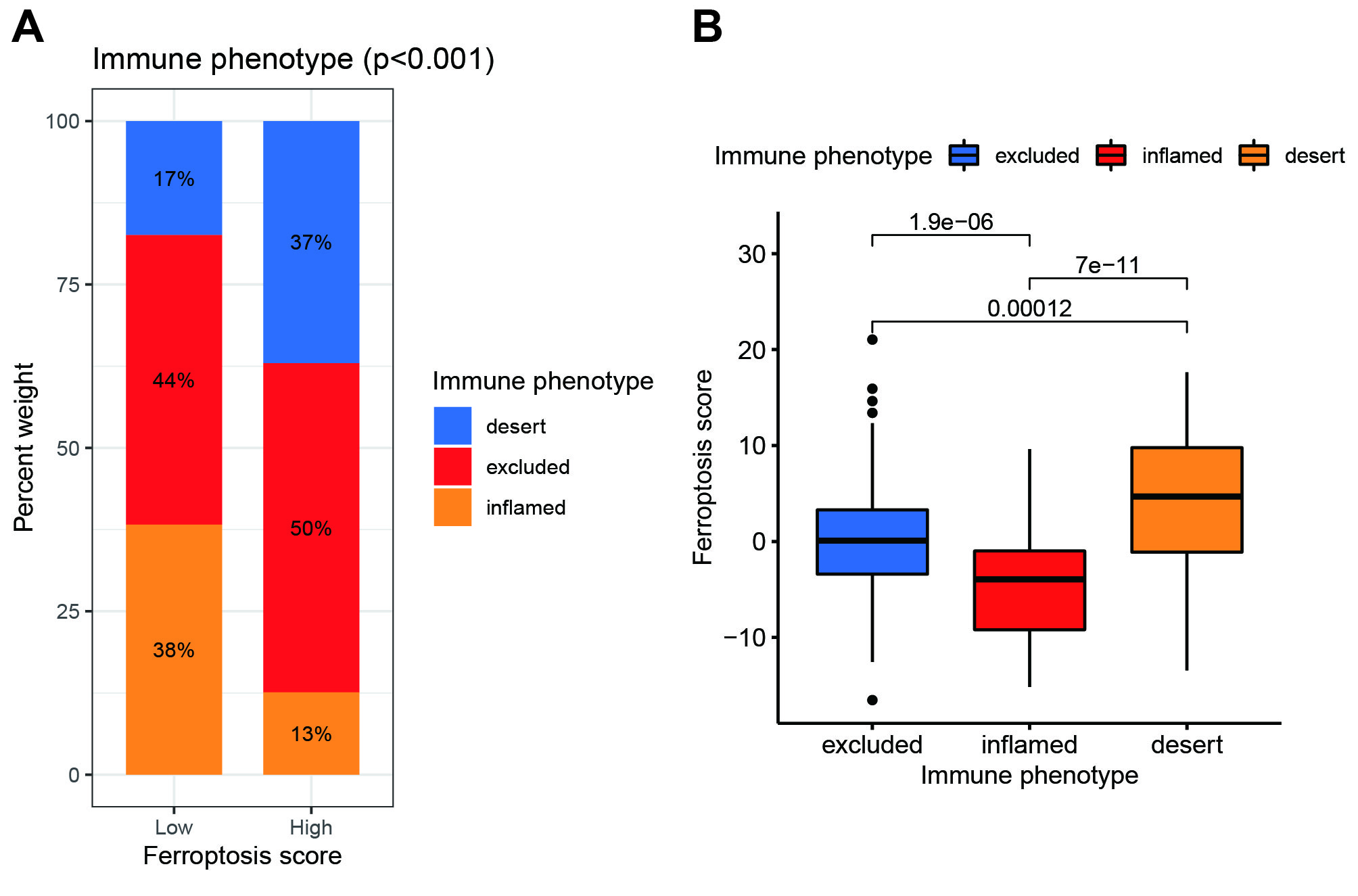

Supplement: Supplementary file 12 [file Image6.JPEG]
