# Supplementary material for: Ferroptosis Patterns and Tumor Microenvironment Infiltration Characterization in Bladder Cancer
Source: Front Cell Dev Biol. 2022 Mar 21;10:832892. doi: 10.3389/fcell.2022.832892 (PMC8978677; doi:10.3389/fcell.2022.832892)
Supplement: Supplementary file 8 [file Table5.DOCX]

**ID Immune phenotype**

SAM4305ab968b90 desert

SAMb2f1d0e54ece desert

SAMd0e47be700b0 desert

SAMd027124354ce desert

SAMc0d625a50eb8 desert

SAM18039827e1b9 desert

SAMc692536a795a desert

SAM468a9e1dc821 desert

SAM6cb230f208a8 desert

SAM9fb814c22bdb desert

SAMbcbc7957c264 desert

SAMe9ae8beb82fa desert

SAM23095936e611 desert

SAM61baf919bb01 desert

SAM9539a4f19ebc desert

SAMeff2ce356ccb desert

SAM110501d0eedb desert

SAMc0ef41aa6c8b desert

SAMe712352fb82a desert

SAMd98bac0a070f desert

SAM8e8ef2368dfa desert

SAMfd947610629d desert

SAM943df5cf15df desert

SAMb3c02294aba7 desert

SAMc0da5d48686d desert

SAM025b45c27e05 desert

SAMe07c4560772d desert

SAMffa5c7cad0e5 desert

SAM4b0175e8db6e desert

SAMf28c01545593 desert

SAM54e58f1b0230 desert

SAMcb132b0cdd2c desert

SAMe50d15fde368 desert

SAM8533e5e261d6 desert

SAM822b226466a1 desert

SAM3e8baff50d7a desert

SAM3ee5dcd894f0 desert

SAM8a1b0e02ee42 desert

SAMa1871f491b02 desert

SAM675a12a09c15 desert

SAM5a2347c0498a desert

SAM28e6031ac18b desert

SAMd86389d0d768 desert

SAM63b2189c36d7 desert

SAM0d855cff64e6 desert

SAM7746b76437e6 desert

SAM548551ef782c desert

SAM5234688806a7 desert

SAM2c9586161ce6 desert

SAM7a9093b9c7e9 desert

SAM8f2275c36e8c desert

SAMfed609955db9 desert

SAMb8070b7937e7 desert

SAMf2aae1443f67 desert

SAM5d989c86255e desert

SAMf82bbdc267c8 desert

SAMd2492b2a31bb desert

SAM491e341d5a82 desert

SAM29da928587ad desert

SAM1dda30f1c5be desert

SAM9daccafc18db desert

SAM25510f300d79 desert

SAMc97f35a29d16 desert

SAMbc8dc3a7b54e desert

SAM14938611a2d3 desert

SAM7edacb3deb65 desert

SAM2b672f4336c7 desert

SAM1ab1b28d9f2b desert

SAM9725303dce0c desert

SAM58e7832f4e7d desert

SAMc57eadb2d82b desert

SAM6792d6e98068 desert

SAM5cc2d9036053 desert

SAM1f83ebd6be9b desert

SAM4918c524b83a desert

SAM6157c8f38b72 desert

SAM698d8d76b934 excluded

SAMc1b27bc16435 excluded

SAMf275eb859a39 excluded

SAM7f0d9cc7f001 excluded

SAMcf018fee2acd excluded

SAM49f9b2e57aa5 excluded

SAM2e7aa8fa0ab3 excluded

SAM36d87392593b excluded

SAM681e4bf7cf85 excluded

SAM557dde1b9f3e excluded

SAMb8f13a0525a6 excluded

SAM23aa15d4a0b0 excluded

SAMb963dda93cfd excluded

SAM7fb6987514a4 excluded

SAMba7176afe070 excluded

SAMbe83eae4026e excluded

SAMe5bc41772bc9 excluded

SAMdb3f50c9129c excluded

SAMbf1a3ae828e6 excluded

SAMd4c0837b0997 excluded

SAMb419a8fcbfcd excluded

SAM17c45bf16bb6 excluded

SAM2070b416069c excluded

SAM0ce9c983b20f excluded

SAM97a00e0929fb excluded

SAM2f228939632f excluded

SAM36851bc8b9ae excluded

SAM297c0301e861 excluded

SAM075e037d95bc excluded

SAM6d2ae0c39b96 excluded

SAM30b5c6c54cf7 excluded

SAM08cce2fa88f2 excluded

SAM39eb94fa504d excluded

SAM4501e41e4751 excluded

SAMd35318127278 excluded

SAMa913c6139ec8 excluded

SAM75142fcab9df excluded

SAMd697ba701077 excluded

SAM166a419a4e5a excluded

SAM2dc578e0165f excluded

SAM52500cabdd36 excluded

SAM14df63a65411 excluded

SAMf3a9bce50099 excluded

SAMdab9ca8fb5de excluded

SAMaec7380f9ab0 excluded

SAMaf7578d55754 excluded

SAMbd8ee73983b8 excluded

SAMe94c30c30616 excluded

SAM5e3bae090b8c excluded

SAM3cb94b0d5297 excluded

SAMe97af0feefdf excluded

SAMdcae54fcd7fa excluded

SAMdad5c29dc105 excluded

SAMaaf505c36f93 excluded

SAM73663ee4a96e excluded

SAM0a7c2091dd56 excluded

SAM99a46b9eec27 excluded

SAM1ac4e3dee297 excluded

SAM3e04eb914f3d excluded

SAMc2a1820d4e6b excluded

SAMabc151b01ea3 excluded

SAMb15ad09d6e24 excluded

SAM961d04c42bd9 excluded

SAM19fec8f3b3bd excluded

SAM7ee2b6e4d6b3 excluded

SAMaff272833538 excluded

SAM415f36ad349e excluded

SAMeb29625f76a5 excluded

SAM30cf07d4874f excluded

SAMd135d5867fe3 excluded

SAMb470eb8f04be excluded

SAM0571f17f4045 excluded

SAM4b7ea015fd9e excluded

SAM18be5b395318 excluded

SAMe9475f77504b excluded

SAMe41b1e773582 excluded

SAM978a587b207e excluded

SAMd3bd67996035 excluded

SAMfddc359e862b excluded

SAM753d4bb52dbe excluded

SAMd3601288319e excluded

SAM59f392864f5d excluded

SAM26104d5adc89 excluded

SAM18a4dabbc557 excluded

SAM2bba8cb35e48 excluded

SAMcee0fa8c05b4 excluded

SAM5c139c5c1c4f excluded

SAMc1251c7bfee2 excluded

SAM85f0a3ac1c45 excluded

SAM27299aed7681 excluded

SAM49d48750e294 excluded

SAM7aa01fc49a80 excluded

SAM0257bbbbd388 excluded

SAMa321770ac31c excluded

SAM3894ac3956a5 excluded

SAM3779e979db6b excluded

SAM1c0ecfb3eb63 excluded

SAMbf91f27e7f9b excluded

SAMbcb07ba81cee excluded

SAM9aa6a095a9d6 excluded

SAM957378bd907f excluded

SAM2624229effe8 excluded

SAM670649e105b5 excluded

SAM8e469834acc1 excluded

SAM8e43e9caf307 excluded

SAM99b1f6a9534e excluded

SAM7d7c54623618 excluded

SAM181b638b8248 excluded

SAMbfdffb97c446 excluded

SAM00b9e5c52da9 excluded

SAM1c8b086175ca excluded

SAM9eebdef2858a excluded

SAMff41c4e8c08f excluded

SAM1a87df750b9d excluded

SAM0f956e757453 excluded

SAMe56c96c51190 excluded

SAM1bcc62d8290c excluded

SAMe0c49ea0df5d excluded

SAM59289ca42c99 excluded

SAM7fb7a13c096b excluded

SAM87a8e18eb45b excluded

SAM1f3c93814cb9 excluded

SAM6ff654a20f98 excluded

SAM65afda25b920 excluded

SAM07a93a28f801 excluded

SAM94859b440b1d excluded

SAMd5ab7fbfab4e excluded

SAM5767dd75d142 excluded

SAM3b1066e5801b excluded

SAM572f19794c96 excluded

SAM1abf01dd4544 excluded

SAM4caabd64e7fd excluded

SAMc6eff056c89a excluded

SAM8b4b8b0f9e73 excluded

SAM85e41e7f33f9 inflamed

SAMdf3e42c8672a inflamed

SAM4edbe45817b3 inflamed

SAM36a9225b0222 inflamed

SAMe7bf6c015192 inflamed

SAM9a2cf3c06fb3 inflamed

SAM7d2dfba6cd84 inflamed

SAM9cafb905b36a inflamed

SAM3f2033c90438 inflamed

SAM032c642382a7 inflamed

SAM8884fe446d20 inflamed

SAMa1e62d323e1d inflamed

SAM1fa6bcb7fc48 inflamed

SAMcabb6d58ff55 inflamed

SAMd636e3461955 inflamed

SAMb4c7a001537d inflamed

SAM2570ff4aae6e inflamed

SAM2dc3f04e45e9 inflamed

SAM9448d858692c inflamed

SAM714285adf612 inflamed

SAM560f23d6a3ad inflamed

SAM61b9d4d84c64 inflamed

SAM73b653ae20d1 inflamed

SAM28687037e4ff inflamed

SAM45c8e6412c66 inflamed

SAMe1eb5d988760 inflamed

SAMe3210d3632b4 inflamed

SAM3f446449bf81 inflamed

SAMb0d11db9aa79 inflamed

SAM6083aac8db99 inflamed

SAMd7d57ee3a863 inflamed

SAM31291c256373 inflamed

SAM9e11ec6bea80 inflamed

SAM065890737112 inflamed

SAM1e9c4d1d39ae inflamed

SAM9306c5c92444 inflamed

SAM6cbc10abddb0 inflamed

SAM203dcf14f927 inflamed

SAM76a431ba6ce1 inflamed

SAMab8052a03398 inflamed

SAMba1a34b5a060 inflamed

SAMfb7aec7cb0e2 inflamed

SAM6780ed436b55 inflamed

SAMd43f8933066b inflamed

SAMc919aebc7fdd inflamed

SAM91c47b054ffb inflamed

SAM5fe7a81a39dd inflamed

SAM12502d970c10 inflamed

SAM9681450bbc90 inflamed

SAM3b15b4c6311d inflamed

SAMeaa477a5384b inflamed

SAM2eb07dedf07f inflamed

SAMbda79f955628 inflamed

SAM9b9d48b0b02c inflamed

SAM47fc46c3d6be inflamed

SAM7829a341b9f3 inflamed

SAMaf42c1541269 inflamed

SAM09c84ec0cf34 inflamed

SAM7538ad9ff524 inflamed

SAM95c70496ffb5 inflamed

SAM771445e92421 inflamed

SAM6662f5181f87 inflamed

SAM18b9351e265a inflamed

SAM5fc9ae0aed1f inflamed

SAMef0e3d2415fd inflamed

SAMcc7a42d87e9c inflamed

SAMce39dd79b441 inflamed

SAM75f12d1a55fc inflamed

SAMf20b827dca51 inflamed

SAM31d9176e11fb inflamed

SAMb8101c538753 inflamed

SAM80c6183220e6 inflamed

SAM1f66db567eb5 inflamed

SAMe7e4f7c076a7 inflamed
