# Supplementary material for: Ferroptosis Patterns and Tumor Microenvironment Infiltration Characterization in Bladder Cancer
Source: Front Cell Dev Biol. 2022 Mar 21;10:832892. doi: 10.3389/fcell.2022.832892 (PMC8978677; doi:10.3389/fcell.2022.832892)
Supplement: Supplementary file 11 [file Table6.DOCX]

**id Ferroptosis score group**

SAMf2ce197162ce -3.32225058160483 Low

SAM698d8d76b934 -7.14028429305707 Low

SAMc1b27bc16435 4.99239493656228 High

SAM85e41e7f33f9 -12.470550134894 Low

SAMf275eb859a39 8.15015416780188 High

SAM7f0d9cc7f001 -0.841755062442368 Low

SAM4305ab968b90 13.9930956826754 High

SAMcf018fee2acd 5.16923868620777 High

SAMb2f1d0e54ece -10.3932504699262 Low

SAMcc4675f394a1 4.49056920206376 High

SAM49f9b2e57aa5 6.26494237531493 High

SAM2e7aa8fa0ab3 3.39007417818152 High

SAMd0e47be700b0 10.0724293879917 High

SAMdf3e42c8672a -8.72186275186545 Low

SAMd027124354ce -8.791522961341 Low

SAM36d87392593b -5.44901754107635 Low

SAM4edbe45817b3 -9.68629812462875 Low

SAM36a9225b0222 -10.2048669457738 Low

SAMe7bf6c015192 -3.39857369252928 Low

SAM6dd7ad1d797d -16.2341043773917 Low

SAMc0d625a50eb8 -1.1829371638786 Low

SAM18039827e1b9 5.87683116765658 High

SAM681e4bf7cf85 3.47660865282607 High

SAMc692536a795a -1.03383141918903 Low

SAM9a2cf3c06fb3 -3.2820095032517 Low

SAM557dde1b9f3e 1.36939904129255 High

SAMb8f13a0525a6 -7.292662282505 Low

SAM23aa15d4a0b0 2.01368880813872 High

SAM468a9e1dc821 -0.918143092773758 Low

SAM81b71522417a 4.82257122038634 High

SAM6cb230f208a8 5.77980123539412 High

SAM0684af734db1 8.64209417865744 High

SAMb963dda93cfd -11.2102327358556 Low

SAMa9ca8536d2b1 -4.06778668622628 Low

SAM9fb814c22bdb -8.693131115031 Low

SAMbcbc7957c264 8.45121144517655 High

SAM7fb6987514a4 -6.55935533536144 Low

SAM63405b04ab2d -5.79045540856307 Low

SAM18bc1078bc15 1.06126722528156 High

SAM7d2dfba6cd84 -2.71028166232088 Low

SAMd1bd63734394 -1.98029068687122 Low

SAMe9ae8beb82fa -2.64798989202223 Low

SAMb0a83e5fbde9 8.1478730817519 High

SAMba7176afe070 14.6318044319446 High

SAMae1690469964 8.75465627254129 High

SAMbe83eae4026e 4.271615324682 High

SAMe5bc41772bc9 -5.06218711934779 Low

SAM23095936e611 9.21542573726589 High

SAM7114d99032ec 2.30122081465284 High

SAMd215b503f99a -4.86547811929916 Low

SAMdb3f50c9129c 1.61112342583405 High

SAMbf1a3ae828e6 -3.99574312239399 Low

SAM52e3fa3ad574 3.34197785232182 High

SAMd4c0837b0997 -2.49134621252618 Low

SAM9cafb905b36a -12.5375543743751 Low

SAM3f2033c90438 -6.52810408532336 Low

SAM032c642382a7 -11.515780842069 Low

SAM5ffd7e4cd794 1.40207417473826 High

SAMb419a8fcbfcd 1.25369150596245 High

SAM8884fe446d20 -15.1678736495958 Low

SAM17c45bf16bb6 13.4063475164634 High

SAM2070b416069c 6.09454346708233 High

SAM0ce9c983b20f -6.21895090148586 Low

SAMa1e62d323e1d 2.80761621753719 High

SAM61baf919bb01 -0.574643149039169 Low

SAM97a00e0929fb 1.89365697684425 High

SAM2f228939632f -4.655629633532 Low

SAM9539a4f19ebc 5.33679292799705 High

SAM36851bc8b9ae 6.32383732411738 High

SAM297c0301e861 -12.5618521491126 Low

SAM1fa6bcb7fc48 9.62236226888496 High

SAM075e037d95bc 15.921660116416 High

SAM6d2ae0c39b96 7.33646915363115 High

SAMcabb6d58ff55 -7.73425289652505 Low

SAM716f54e468f4 -0.672740630221423 Low

SAMeff2ce356ccb 8.70695170020775 High

SAM110501d0eedb 2.99359493746233 High

SAM2e9ac0b1b250 -5.04436406569726 Low

SAMc0ef41aa6c8b 11.2437042414954 High

SAMd636e3461955 -7.63828613876127 Low

SAMe712352fb82a 4.62234667142616 High

SAM30b5c6c54cf7 -2.81379749621739 Low

SAMd98bac0a070f 0.893780258728646 High

SAM8e8ef2368dfa -1.36568959656911 Low

SAM08cce2fa88f2 9.4199926113906 High

SAMb4c7a001537d 7.9320625175225 High

SAMfd947610629d -2.3979306845929 Low

SAM943df5cf15df -8.05714681545992 Low

SAM31f41dd0d6ca -1.76144230488866 Low

SAMb3c02294aba7 -0.953753515587573 Low

SAM39eb94fa504d -5.31281090102967 Low

SAM62fb1388c871 2.32432239417039 High

SAMc0da5d48686d -9.43134777127254 Low

SAMe7bcab05402e -1.12706911913107 Low

SAM2570ff4aae6e -3.76409357203344 Low

SAM2dc3f04e45e9 -1.9282307244057 Low

SAM9448d858692c -3.41623280665546 Low

SAM4501e41e4751 -6.94733244243771 Low

SAM714285adf612 -9.42280489173083 Low

SAM5b57e47fdcb3 6.05345314002305 High

SAMae4da274eded 0.182224759973833 High

SAMd35318127278 4.56257932974208 High

SAMa913c6139ec8 -2.66512788789313 Low

SAM75142fcab9df 0.922332552139133 High

SAMd697ba701077 5.24059991718896 High

SAM166a419a4e5a -3.31662760430064 Low

SAM025b45c27e05 -6.66374140626706 Low

SAMe07c4560772d 13.7710909947123 High

SAM2dc578e0165f -6.5512802713605 Low

SAM3785587846ce 12.3884476069526 High

SAM560f23d6a3ad -4.34541040047115 Low

SAM52500cabdd36 7.53735927629697 High

SAM14df63a65411 1.78403518443999 High

SAMf3a9bce50099 -1.00647698086072 Low

SAMffa5c7cad0e5 -6.59508172887113 Low

SAMdab9ca8fb5de 2.73980262676955 High

SAM34430ef08e5b 2.46176668979129 High

SAM4b0175e8db6e 1.5352089456198 High

SAMf28c01545593 17.6329994563371 High

SAMaec7380f9ab0 -0.0805395946431071 Low

SAMaf7578d55754 -3.349834668389 Low

SAMbd8ee73983b8 2.42807461067467 High

SAMe94c30c30616 -1.07077157669296 Low

SAM54e58f1b0230 7.71679496152363 High

SAM5e3bae090b8c 2.79672782932014 High

SAM3cb94b0d5297 0.369634835710335 High

SAM61b9d4d84c64 9.07829186967816 High

SAM8a42c0d59187 5.31789192144949 High

SAMcb132b0cdd2c -1.19127008559712 Low

SAMe97af0feefdf 1.83310863098378 High

SAMee3844cc0b9f -4.43719451154442 Low

SAM73b653ae20d1 -15.0762504885984 Low

SAMdcae54fcd7fa -0.839700782647574 Low

SAMdad5c29dc105 3.02844323073026 High

SAMa424c75831b4 0.266944967849606 High

SAM28687037e4ff -10.4966163178171 Low

SAMe50d15fde368 12.2405847671921 High

SAM45c8e6412c66 -9.03641359560544 Low

SAM8533e5e261d6 9.67603265835312 High

SAMaaf505c36f93 6.43383954288844 High

SAM73663ee4a96e 4.51179479254344 High

SAM0bdb3428bd13 -2.69523151347666 Low

SAM3330c03fdf00 0.0876772332123625 High

SAM0a7c2091dd56 -12.3425457290687 Low

SAM7bff231634e9 -5.74586564203323 Low

SAM5d1dfd5207f5 10.5124038099349 High

SAMeb587a68006b -3.53963223794001 Low

SAM99a46b9eec27 -2.00222430398149 Low

SAM1ac4e3dee297 12.3184271872518 High

SAM553c3c35b847 -8.32479681061903 Low

SAM3e04eb914f3d -2.27652621776244 Low

SAMc2a1820d4e6b 0.616736877554418 High

SAM822b226466a1 15.8059773266863 High

SAMabc151b01ea3 -0.739798973279053 Low

SAMe1eb5d988760 -2.06207261206039 Low

SAMe3210d3632b4 -13.2499289927286 Low

SAMb15ad09d6e24 -9.93282095257478 Low

SAM7893196e0e89 -1.71083960652189 Low

SAM3f446449bf81 0.999356436315131 High

SAM961d04c42bd9 3.57817818595542 High

SAMb0d11db9aa79 4.69376157266638 High

SAM19fec8f3b3bd -10.6659359391589 Low

SAM6083aac8db99 -3.27201310114543 Low

SAM7ee2b6e4d6b3 9.23450969808224 High

SAMa90d73f8d891 -2.01253298487314 Low

SAMaff272833538 -2.46319899538524 Low

SAM415f36ad349e -7.59732442660851 Low

SAM7c67b05aa109 13.8330199727771 High

SAMad83c9c53537 -1.67171967313019 Low

SAM3e8baff50d7a 2.77510438649249 High

SAM3ee5dcd894f0 1.77492463748735 High

SAM8a1b0e02ee42 2.05120883163707 High

SAMd7d57ee3a863 -8.69820184899297 Low

SAMeb29625f76a5 6.16117615882 High

SAM563d6233dfa2 2.11124661673131 High

SAM4581bac493af 0.103215223715249 High

SAMa1871f491b02 9.57671085314496 High

SAM30cf07d4874f -4.9354992334053 Low

SAM31291c256373 -2.86280089011376 Low

SAMd135d5867fe3 -16.5304539462659 Low

SAM9e11ec6bea80 -10.4427420148522 Low

SAM065890737112 8.57623138290128 High

SAMb470eb8f04be 2.58444963251411 High

SAM675a12a09c15 4.16619445449748 High

SAM1e9c4d1d39ae -0.739525453065493 Low

SAM5a2347c0498a 8.58445519711129 High

SAM0571f17f4045 0.866014182813639 High

SAM4b7ea015fd9e 21.0418546312191 High

SAM9306c5c92444 3.69286919118807 High

SAMb15ac6e4c4ef 7.29327033221367 High

SAM28e6031ac18b 11.4531865568906 High

SAMd86389d0d768 13.7787638500731 High

SAM63b2189c36d7 13.6565329133912 High

SAM18be5b395318 2.97923381385467 High

SAM0d855cff64e6 0.200380977572376 High

SAM6cbc10abddb0 -9.1970655713673 Low

SAMdee1011782cd -1.76789633495786 Low

SAMe9475f77504b -4.24248142355191 Low

SAMa535fcdf18a0 0.508813608103191 High

SAM7746b76437e6 10.252169548638 High

SAM548551ef782c -2.28497498959618 Low

SAM203dcf14f927 -1.27212287538899 Low

SAMe41b1e773582 -1.75471291245173 Low

SAM978a587b207e -8.08456766653181 Low

SAM5234688806a7 11.9664679866396 High

SAM2c9586161ce6 10.7681884216294 High

SAM76a431ba6ce1 -3.8685965781219 Low

SAM7a9093b9c7e9 -1.08617603427349 Low

SAM8f2275c36e8c 7.52381383497337 High

SAMab8052a03398 -6.42969260316974 Low

SAMd3bd67996035 1.85905357118292 High

SAMfddc359e862b 1.65644847812932 High

SAM753d4bb52dbe -0.471984813162079 Low

SAMd3601288319e 1.22537388655997 High

SAM59f392864f5d -2.80179543603097 Low

SAM26104d5adc89 0.189028015938724 High

SAMba1a34b5a060 -6.86960572998371 Low

SAM18a4dabbc557 -0.366388803865161 Low

SAMfb7aec7cb0e2 -9.18428181511629 Low

SAMfed609955db9 -1.39764694329948 Low

SAMb8070b7937e7 12.1483631030022 High

SAMf2aae1443f67 7.75138635089461 High

SAM2bba8cb35e48 -2.04084349449734 Low

SAMcee0fa8c05b4 2.70984817205576 High

SAM5c139c5c1c4f 4.12386881128234 High

SAMc1251c7bfee2 -8.43010952716633 Low

SAM6780ed436b55 -5.57386962312384 Low

SAM85f0a3ac1c45 10.1507504938107 High

SAM9d2494119c05 6.4386930790046 High

SAM9410b866974a -7.48452515522583 Low

SAM5d989c86255e -3.02512951399852 Low

SAM27299aed7681 10.5524102516038 High

SAM49d48750e294 -2.56138886698673 Low

SAMd43f8933066b 0.180555748648129 High

SAM7aa01fc49a80 -0.942781758680304 Low

SAM0257bbbbd388 4.252255667046 High

SAMc919aebc7fdd 2.41817517983008 High

SAMa321770ac31c -1.68349618624161 Low

SAM3894ac3956a5 1.52700806960542 High

SAM3779e979db6b 2.43313058917455 High

SAM1c0ecfb3eb63 -8.26139583288613 Low

SAMbf91f27e7f9b 0.227474894159474 High

SAMbcb07ba81cee -1.14947830014396 Low

SAM9aa6a095a9d6 0.281809880574134 High

SAM957378bd907f 0.928536391944496 High

SAMf82bbdc267c8 12.0407235140394 High

SAM2624229effe8 -0.417395407554444 Low

SAMd2492b2a31bb -13.4286524006858 Low

SAM670649e105b5 -4.47614753869699 Low

SAM91c47b054ffb 2.76394819860886 High

SAM491e341d5a82 2.25487899185458 High

SAM5fe7a81a39dd 3.55879839558931 High

SAM29da928587ad 2.29094207770729 High

SAM0a0f2bac4b20 -8.66720962148481 Low

SAM8e469834acc1 4.34445530427159 High

SAM8e43e9caf307 4.95741535328803 High

SAM99b1f6a9534e 2.98763425615439 High

SAM12502d970c10 2.71487032992481 High

SAM9681450bbc90 -4.53767317249122 Low

SAM7d7c54623618 -3.42400822821204 Low

SAM181b638b8248 -8.77120700365725 Low

SAMbfdffb97c446 -7.76333289208708 Low

SAM3b15b4c6311d -11.413317037785 Low

SAM00b9e5c52da9 -5.35588009404607 Low

SAM59b825252c0d -7.13898347105266 Low

SAM1c8b086175ca -5.31987205917407 Low

SAMeaa477a5384b 4.2469330900755 High

SAM1dda30f1c5be -5.66287652271444 Low

SAM9eebdef2858a -0.457629491677659 Low

SAM9daccafc18db -1.8623354405669 Low

SAM2eb07dedf07f -4.57525138050824 Low

SAM25510f300d79 -8.53650362691085 Low

SAMbda79f955628 -1.35068239296223 Low

SAM9b9d48b0b02c 4.81552579321809 High

SAMff41c4e8c08f 5.65267505722925 High

SAMc97f35a29d16 4.91235494822347 High

SAM187e056d6a2a 0.901630065891977 High

SAMbc8dc3a7b54e 7.06964985155615 High

SAM7b40007f4aa4 1.99058771413456 High

SAM568ce160abd9 0.805260652950228 High

SAM14938611a2d3 4.88332932832238 High

SAM1a87df750b9d -8.25733721382082 Low

SAMae02629a97f7 4.50532947170164 High

SAM47fc46c3d6be -9.68636132238848 Low

SAM7829a341b9f3 -3.62191439897093 Low

SAM0f956e757453 -1.40931980084212 Low

SAMaf42c1541269 -5.647678189095 Low

SAM09c84ec0cf34 -3.16515129565385 Low

SAM7538ad9ff524 -0.90158153244923 Low

SAM95c70496ffb5 -3.33441189156436 Low

SAM7edacb3deb65 4.7505069083356 High

SAMb2e4a082541a 2.40626937718276 High

SAM771445e92421 1.08274947046188 High

SAM59fda9035d1d -8.08032504480345 Low

SAM727c0e92a2a7 -3.93766946399864 Low

SAM6f2a102a99df -7.3775196463913 Low

SAMe56c96c51190 -10.3667623651763 Low

SAM2b672f4336c7 6.48006584612175 High

SAM1ab1b28d9f2b 2.01555580992009 High

SAM1bcc62d8290c -0.0860721726815692 Low

SAM6662f5181f87 -1.35197988338295 Low

SAMe0c49ea0df5d -2.21852699272379 Low

SAM59289ca42c99 -8.21309362591095 Low

SAM18b9351e265a -5.37444131204338 Low

SAM7fb7a13c096b 0.0967471718974147 High

SAM87a8e18eb45b 9.79997541375272 High

SAM1f3c93814cb9 -1.20136510333999 Low

SAM5fc9ae0aed1f -13.5068473897674 Low

SAM9725303dce0c 6.92115028751826 High

SAM6ff654a20f98 0.740024333400602 High

SAM65afda25b920 -8.56220170605479 Low

SAMef0e3d2415fd -4.01974118088499 Low

SAM07a93a28f801 1.45650797935631 High

SAM94859b440b1d 7.32686143296378 High

SAM58e7832f4e7d 16.156323098103 High

SAMc57eadb2d82b 10.3976624603828 High

SAMd5ab7fbfab4e -0.342836249221709 Low

SAM5767dd75d142 -0.706727820936174 Low

SAMaabf4afe4213 0.989912476176413 High

SAMcc7a42d87e9c 4.35313741743837 High

SAMce39dd79b441 -7.30504842940737 Low

SAM75f12d1a55fc -4.62666418855211 Low

SAM6964a6d7b967 3.81242068108862 High

SAM3b1066e5801b 0.511642025728222 High

SAMf20b827dca51 -3.19241257982863 Low

SAM6792d6e98068 13.325108477322 High

SAM31d9176e11fb -2.53846578583632 Low

SAM04c589eb3fb3 -2.58705204934835 Low

SAM5cc2d9036053 -0.430528427728043 Low

SAMb8101c538753 -9.65395304022098 Low

SAM80c6183220e6 -10.6926415797109 Low

SAM572f19794c96 -4.96118510152933 Low

SAM1abf01dd4544 -2.61640073132545 Low

SAM1f83ebd6be9b 10.1903625818414 High

SAM1f66db567eb5 -11.7403028356539 Low

SAM4918c524b83a 5.1185418043193 High

SAMa0ca029b7afd 1.33286652801807 High

SAM6157c8f38b72 -0.342396748876205 Low

SAMe7e4f7c076a7 -14.5489858134815 Low

SAMbe25e2c88f3e -1.40589655486561 Low

SAM4caabd64e7fd 5.7650513647205 High

SAMc6eff056c89a 2.67611459048242 High

SAM5cfa1699bdb7 -2.47258270255396 Low

SAMda4d892fddc8 0.154204163164815 High

SAM3a1c9632ff7b 6.28790336950583 High

SAM8b4b8b0f9e73 0.0657110367456673 High

SAMe3d4266775a9 4.93183560015515 High

SAM2de7cffb5f72 2.74004152186128 High
